# Multi-cohort machine learning identifies predictors of cognitive impairment in Parkinson’s disease

**DOI:** 10.1038/s41746-025-01862-1

**Published:** 2025-07-26

**Authors:** Rebecca Ting Jiin Loo, Lukas Pavelka, Graziella Mangone, Fouad Khoury, Marie Vidailhet, Jean-Christophe Corvol, Enrico Glaab, On behalf of the NCER-PD Consortium, Geeta Acharya, Gloria Aguayo, Myriam Alexandre, Muhammad Ali, Wim Ammerlann, Giuseppe Arena, Michele Bassis, Roxane Batutu, Katy Beaumont, Sibylle Béchet, Guy Berchem, Alexandre Bisdorff, Ibrahim Boussaad, David Bouvier, Lorieza Castillo, Gessica Contesotto, Nancy De Bremaeker, Brian Dewitt, Nico Diederich, Rene Dondelinger, Nancy E. Ramia, Maria Fernanda Niño Uribe, Angelo Ferrari, Ana Festas Lopes, Katrin Frauenknecht, Joëlle Fritz, Carlos Gamio, Manon Gantenbein, Piotr Gawron, Laura Georges, Soumyabrata Ghosh, Marijus Giraitis, Enrico Glaab, Martine Goergen, Elisa Gómez De Lope, Jérôme Graas, Mariella Graziano, Valentin Groues, Anne Grünewald, Gaël Hammot, Anne-Marie Hanff, Linda Hansen, Michael Heneka, Estelle Henry, Margaux Henry, Sylvia Herbrink, Sascha Herzinger, Alexander Hundt, Nadine Jacoby, Sonja Jónsdóttir, Jochen Klucken, Olga Kofanova, Rejko Krüger, Pauline Lambert, Zied Landoulsi, Roseline Lentz, Victoria Lorentz, Tainá M. Marques, Guilherme Marques, Patricia Martins Conde, Patrick May, Deborah Mcintyre, Chouaib Mediouni, Francoise Meisch, Alexia Mendibide, Myriam Menster, Maura Minelli, Michel Mittelbronn, Saïda Mtimet, Maeva Munsch, Romain Nati, Ulf Nehrbass, Sarah Nickels, Beatrice Nicolai, Jean-Paul Nicolay, Fozia Noor, Clarissa P. C. Gomes, Sinthuja Pachchek, Claire Pauly, Laure Pauly, Lukas Pavelka, Magali Perquin, Achilleas Pexaras, Armin Rauschenberger, Rajesh Rawal, Dheeraj Reddy Bobbili, Lucie Remark, Ilsé Richard, Olivia Roland, Kirsten Roomp, Eduardo Rosales, Stefano Sapienza, Venkata Satagopam, Sabine Schmitz, Reinhard Schneider, Jens Schwamborn, Raquel Severino, Amir Sharify, Ruxandra Soare, Ekaterina Soboleva, Kate Sokolowska, Maud Theresine, Hermann Thien, Elodie Thiry, Rebecca Ting Jiin Loo, Johanna Trouet, Olena Tsurkalenko, Michel Vaillant, Carlos Vega, Liliana Vilas Boas, Paul Wilmes, Evi Wollscheid-Lengeling, Gelani Zelimkhanov

**Affiliations:** 1 Biomedical Data Science Group, Luxembourg Centre for Systems Biomedicine (LCSB), University of Luxembourg, Esch-sur-Alzette, Luxembourg; 2 Transversal Translational Medicine, Luxembourg Institute of Health (LIH), Strassen, Luxembourg; 3 Sorbonne Université, Paris Brain Institute - ICM, Inserm, CNRS, Assistance Publique Hôpitaux de Paris, Pitié-Salpêtrière Hospital, Department of Neurology, Paris, France; 4 Luxembourg Institute of Health (LIH), Strassen, Luxembourg; 5 Luxembourg Centre for Systems Biomedicine (LCSB), University of Luxembourg, Esch-sur-Alzette, Luxembourg; 6 Centre Hospitalier de Luxembourg, Strassen, Luxembourg; 7 Centre Hospitalier Emile Mayrisch, Esch-sur-Alzette, Luxembourg; 8 Laboratoire National de Santé, Dudelange, Luxembourg; 9 Association of Physiotherapists in Parkinson’s Disease Europe, Esch-sur-Alzette, Luxembourg; 10 Faculty of Science, Technology and Medicine, University of Luxembourg, Esch-sur-Alzette, Luxembourg; 11 Department of Epidemiology, CAPHRI School for Public Health and Primary Care, Maastricht University Medical Centre, Maastricht, the Netherlands; 12 Private practice, Ettelbruck, Luxembourg; 13 Parkinson Luxembourg Association, Leudelange, Luxembourg; 14 Luxembourg Center of Neuropathology, Dudelange, Luxembourg; 15 Department of Life Sciences and Medicine, University of Luxembourg, Esch-sur-Alzette, Luxembourg; 16 Private practice, Luxembourg, Luxembourg; 17 Pitié-Salpêtrière Hospital, Department of Neurology, Paris, France; 18 La Timone Hospital, Marseille, France; 19 ICM, Paris, France; 20 CEA, Saclay, France; 21 Avicenne Hospital, Bobigny, France; 22 Telecom Sud Paris, Evry, France; 23 Pierre and Marie Curie University, Paris, France; 24 Supelec, Gif-sur-Yvette, France

**Keywords:** Machine learning, Parkinson's disease

## Abstract

Cognitive impairment is a frequent complication of Parkinson’s disease (PD), affecting up to half of newly diagnosed patients. To improve early detection and risk assessment, we developed machine learning models using clinical data from three independent PD cohorts, which are (LuxPARK, PPMI, ICEBERG). Models were trained to predict mild cognitive impairment (*PD-MCI*) and subjective cognitive decline (*SCD*) using Explainable Artificial Intelligence (XAI) for classification and time-to-event analysis. Multi-cohort models showed greater performance stability over single-cohort models, while retaining competitive average performance. Age at diagnosis and visuospatial ability were identified as key predictors. Significant sex differences observed highlight the importance of considering sex-specific factors in cognitive assessment. Men were more likely to report *SCD*. Our findings highlight the potential of multi-cohort machine learning for early identification and personalized management of cognitive decline in PD.

## Introduction

Parkinson’s disease (PD) includes motor and non-motor symptoms, the latter comprising a wide range of neuropsychiatric, autonomic, and sensory disturbances. Cognitive impairment (CI) affects 20–50% of newly diagnosed patients and significantly impacts quality of life^1–3^. It can manifest early in the disease course, with deficits in several domains, including memory, attention, executive function, visuospatial skills, and language^4,5^. CI in PD spans from mild cognitive impairment (*PD-MCI*) to PD dementia, with *PD-MCI* representing a key stage for potential early therapeutic intervention strategies.

Early identification of high-risk patients for development of CI is essential for preventive strategies, including pharmacological (e.g., cholinesterase inhibitors^6^, medication adjustments^7^) and non-pharmacological options (e.g., cognitive training^8^). While the impact of current interventions remains limited^9,10^, accurate risk prediction can facilitate the development of more targeted treatments and long-term care^3,11^.

CI in PD is influenced by multiple factors, including age at onset^3,12^, motor severity^1,13^, and non-motor symptoms (e.g., depression, apathy, autonomic dysfunction)^14^. The Montreal Cognitive Assessment (MoCA) is a widely used screening tool for assessing cognitive function^3^, with scores between 21 and 25 indicating *PD-MCI* according to Level I Movement Disorder Society (MDS) Task Force criteria^15^, and scores ≤20 suggesting more severe impairment. However, despite its widespread use, MoCA presents several important limitations as a cognitive assessment tool. It provides only a single-time-point measurement that cannot adequately capture the fluctuating nature of cognitive deficits in PD, and lacks sensitivity for detecting subtle or early-stage impairments^16,17^ (particularly in highly educated individuals). Additionally, subjective cognitive decline (*SCD*) provides insights into impairments that may even precede objectively measurable deficits^18^. Although the MoCA is a widely utilized tool for evaluating CI in PD, its correlation with *SCD* remains unclear^18^. Prior studies indicate objective cognitive assessments may not fully align with patient-perceived cognitive difficulties^19^, and *SCD* may be influenced by mood disturbances^20^, sleep disorders^21^, and fatigue^22^. Nevertheless, the concurrent assessment of objective and subjective CI has the potential to provide complementary insights into the patient experience and underlying pathology.

Previous studies on CI in PD focused on single cohorts with small sample sizes and several cohort-specific characteristics^3,23–25^. This cohort-specificity limits the generalizability of their findings and hinders the development of broadly applicable clinical tools. While machine learning (ML) can uncover complex relationships in clinical data, for CI assessment in PD it has only been used in single-cohort studies, limiting model generalizability and applicability across diverse patient groups^26^. To address this, this study integrates clinical data from three independent cohorts (the Luxembourgish Parkinson’s Study (LUXPARK)^27^, the Parkinson’s Progression Markers Initiative (PPMI)^28^, and the French cohort ICEBERG^29^) to identify more robust and generalizable predictors of CI in PD. We predict *PD-MCI* and *SCD* within four years and until the end of the follow-up period, and assess the robustness of models in heterogeneous populations that differ in demographics, disease severity, and follow-up duration by validating them across multiple independent cohorts. Using a cross-cohort modeling approach, we identified the most consistent and reliable predictors that are broadly applicable across different PD populations and might help to inform personalized intervention studies for PD patients at risk of CI in diverse clinical settings.

## Results

### Individual cohort analyses

The performance of ML models for predicting *PD-MCI* and *SCD* was first evaluated in each cohort separately (LuxPARK, PPMI, and ICEBERG). Here, we focus on presenting the results for the hold-out test set (for detailed cross-validation (CV) results, see Supplementary Tables 1–4).

For *PD-MCI* classification, the model trained and validated on the LuxPARK cohort reached the highest hold-out AUC (0.70), with a cross-validated AUC (CV-AUC) of 0.70 (Supplementary Table 1). In PPMI, the models showed comparable performance (hold-out AUC of 0.69, CV-AUC of 0.70). Performance was lower in ICEBERG due to its smaller sample size (Supplementary Fig. 1).

For time-to-*PD-MCI* analysis, the model derived from the LuxPARK cohort achieved a moderate hold-out C-index of 0.63 (Supplementary Table 2). The PPMI-specific models performed better (hold-out C-index of 0.72). The models’ performance using ICEBERG was again lower, likely due to sample size constraints (Supplementary Fig. 2).

Regarding *SCD* classification, the model using LuxPARK achieved a moderate hold-out AUC of 0.63. The PPMI model performed better (hold-out AUC of 0.70), and the ICEBERG model showed lower performance in line with smaller sample sizes (Supplementary Table 3 and Supplementary Fig. 3).

Furthermore, in the time-to-*SCD* analysis, the model trained on the PPMI cohort achieved the highest performance with a hold-out C-index of 0.76. The model obtained from LuxPARK had a lower hold-out C-index of 0.71, while performance was lowest for the model using ICEBERG (hold-out C-index of 0.60, Supplementary Table 4 and Supplementary Fig. 4).

Both *PD-MCI* and *SCD* analyses identified age at PD diagnosis and baseline MoCA^30^ as most informative among the top-15 predictors (Table 1). Key *PD-MCI* predictors included Benton Judgment of Line Orientation (JLO)^31^ and baseline CI (Movement Disorder Society-Unified Parkinson’s Disease Rating Scale (MDS-UPDRS) Part I^18^). For *SCD*, predictors included MDS-UPDRS Part I and II total scores, Scales for Outcomes in Parkinson’s Disease - Autonomic Dysfunction (SCOPA-AUT) symptoms (particularly gastrointestinal and urinary)^32^, and disease duration.

**Table 1:** Average percentage of predictors selected in 5-fold cross-validation for classification and time-to-event analyses across LuxPARK, PPMI, and ICEBERG cohorts

Mild cognitive impairment (*PD-MCI*)			Subjective cognitive decline (*SCD*)		
Predictors	(1)	(2)	Predictors	(1)	(2)
MDS-UPDRS II - Getting out of bed, car, or deep chair	–	80	SCOPA-AUT Gastrointestinal (GI)	53.3	93.3
MDS-UPDRS I - Urinary problems	–	73.3	MDS-UPDRS Part I score	46.7	100
**Age at PD diagnosis**	73.3	66.7	**Age at PD diagnosis**	40	100
MDS-UPDRS II - Saliva and drooling	40	66.7	Height (cm)	40	100
MDS-UPDRS II - Chewing and swallowing	–	53.3	Weight (kg)	46.7	93.3
**MoCA score (adjusted for education)**	60	33.3	Disease duration since PD diagnosis (years)	40	93.3
MDS-UPDRS I - Pain and other sensations	13.3	80	MDS-UPDRS Part II score	33.3	100
Benton Judgment of Line Orientation	26.7	66.7	SCOPA-AUT Sexual dysfunction	–	66.7
MDS-UPDRS III - Posture (ON)	26.7	66.7	SCOPA-AUT Urinary	26.7	100
Family history of PD	–	46.7	MDS-UPDRS II - Handwriting	40	80
MDS-UPDRS I - Depressed moods	–	46.7	**MoCA score (adjusted for education)**	–	60
MDS-UPDRS I - Cognitive impairment	20	66.7	MDS-UPDRS III - Global spontaneity of movement (ON)	–	60
MDS-UPDRS I - Fatigue	20	60	Tremor	–	60
MDS-UPDRS I - Sleep problems (night)	20	60	BMI (kg/m^2^)	40	73.3
MDS-UPDRS I - Constipation problems	20	60	MDS-UPDRS I - Apathy	26.7	86.7

Statistics on the average percentage of times predictors were selected during 5-fold cross-validation (CV) analyses. It compares data for mild cognitive impairment and subjective cognitive decline (1) classification and (2) time-to-event analyses across the LuxPARK, PPMI, and ICEBERG cohorts. The information presented includes the average percentage of times each feature was chosen in 5-fold CV for single-cohort analyses in LuxPARK, PPMI, and ICEBERG for both classification and time-to-event analyses across all cohorts. Features are listed in descending order based on their average selection percentages in classification and time-to-event analyses, with the top 15 features presented. The predictors shown in bold were consistently selected for mild cognitive impairment and subjective cognitive decline, respectively.

### Multi-cohort analyses

Multi-cohort analyses were conducted to improve model robustness and overcome single-cohort limitations, assessing hold-out test set performance (for more detailed statistics, including cross-validation results, see Supplementary Figs. 1–4 and Supplementary Tables 5–8).

In *PD-MCI* classification, cross-cohort modeling achieved a largest hold-out AUC of 0.67, comparable to the best single-cohort results (Supplementary Table 5). In Leave-ICEBERG-out analyses, the models showed indications of overfitting, with low test set performance (best hold-out performance using GBoost: AUC 0.60). Leave-PPMI-out and Leave-LuxPARK-out analyses performed similarly to cross-cohort analysis (hold-out AUCs of 0.63 and 0.65, respectively).

Cross-cohort modeling for time-to-*PD-MCI* analysis yielded moderate performance, with a largest hold-out C-index of 0.65, similar to the LuxPARK and PPMI single-cohort analyses (Supplementary Table 6). The Leave-ICEBERG-out setting generally resulted in lower performance, except for the CW-GBoost model (hold-out C-index 0.63, CV-C 0.65). Leave-PPMI-out and Leave-LuxPARK-out analyses performed similarly to cross-cohort analysis, showing no trade-off in predictive power for increased robustness.

The cross-cohort analysis in *SCD* classification achieved a hold-out AUC of 0.72, slightly outperforming single-cohort analyses (Supplementary Table 7). Leave-ICEBERG-out showed lower performance (GOSDT-GUESSES hold-out AUC: 0.61, CV-AUC: 0.65), and Leave-LuxPARK-out analysis performed slightly lower (hold-out AUC 0.63, CV-AUC 0.68). Leave-PPMI-out achieved a similar hold-out AUC to the cross-cohort analysis (0.71).

For time-to-*SCD* analysis, the cross-cohort analysis yielded a hold-out C-index of 0.72, similar to the PPMI’s single-cohort results (Supplementary Table 8). Leave-ICEBERG-out achieved a lower hold-out C-index (0.64). Leave-PPMI-out and Leave-LuxPARK-out analyses showed comparable results.

Apart from achieving significant predictive accuracy, model stability is a further important aspect for developing reliable predictive tools that can be further developed towards clinical applications. Multi-cohort models provided more stable performance statistics than single-cohort models across CV cycles (see Supplementary Figs. 5–8). Incorporating diverse populations improved model robustness, reducing cohort-specific biases, and increasing clinical prediction reliability. The lower stability in ICEBERG, explained by its smaller sample size, highlights the importance of statistical power.

In summary, multi-cohort models achieved comparable performance to single-cohort models, with improved model stability and robustness, despite the more challenging nature of prediction tasks across multiple cohorts. This confirms that integrating data across cohorts improves model applicability and reliability while maintaining performance levels.

### Comparative evaluation of cross-study normalization approaches

We assessed cross-study normalization methods by comparing the performance metrics on the hold-out test set data for the normalized and unnormalized models with the highest cross-validated AUC/C-index (CV-AUC/CV-C) in multi-cohort analyses. Normalization improved predictive performance for *PD-MCI* and *SCD* classification and time-to-*SCD* (Supplementary Tables 9, 10).

A notable gain in the Leave-PPMI-out analysis likely reflects distinctive value distributions in PPMI (Supplementary Table 11). However, benefits varied across studies, indicating that normalization can enhance model performance but should be tailored to cohort-specific biases.

### Associations of clinical characteristics with cognitive impairment

**Fig. 1:**
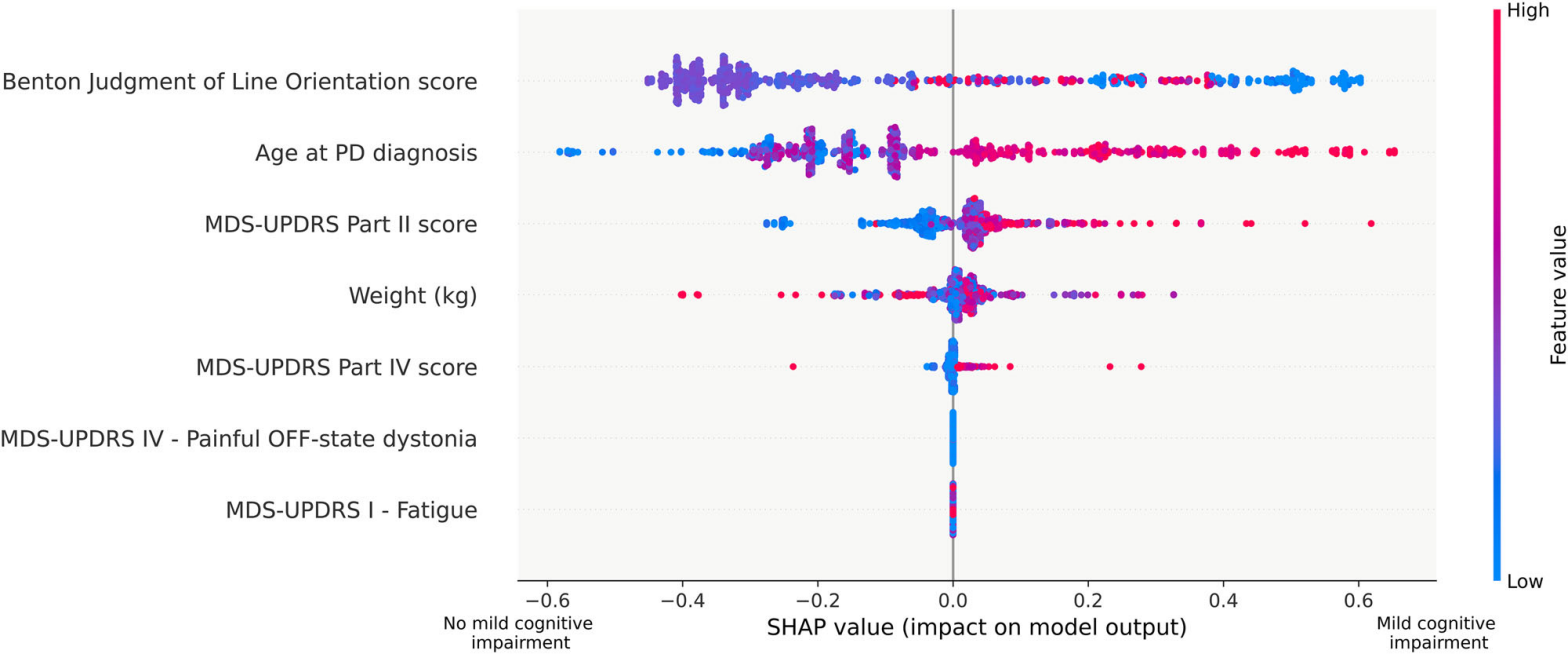
SHAP value plot revealing key predictors’ influence on *PD-MCI* classification in the cross-cohort analysis. Each row shows a predictor’s impact on mild cognitive impairment (*PD-MCI*) classification, with SHAP values indicating the direction and magnitude of effect. Points represent individual patients, with colors indicating the predictor’s value (red = high, blue = low). Positive SHAP values (right side) indicate increased likelihood of *PD-MCI*, while negative values (left side) suggest decreased likelihood. The Benton Judgment of Line Orientation score shows the strongest effect, with lower scores (blue) associated with increased *PD-MCI* risk. Age at PD diagnosis demonstrates the second strongest impact, with later onset (red) correlating with higher *PD-MCI* probability. Additional predictors include MDS-UPDRS subscores (Parts II, IV, and I) and weight, each showing varying degrees of influence on cognitive impairment classification.

Cross-cohort analyses for *PD-MCI* classification and time-to-*PD-MCI* prediction revealed consistent key predictors of CI in PD through SHapley Additive exPlanations (SHAP) value plots (Fig. 1 and Supplementary Fig. 9).

Visuospatial ability (Benton JLO^4^) emerged as a top predictor for *PD-MCI* and time-to-*PD-MCI*, with better performance associated with a lower *PD-MCI* risk and delayed onset. Age at PD diagnosis and advanced motor impairment (MDS-UPDRS Part II and IV^33^) were also associated with increased *PD-MCI* risk.

**Fig. 2:**
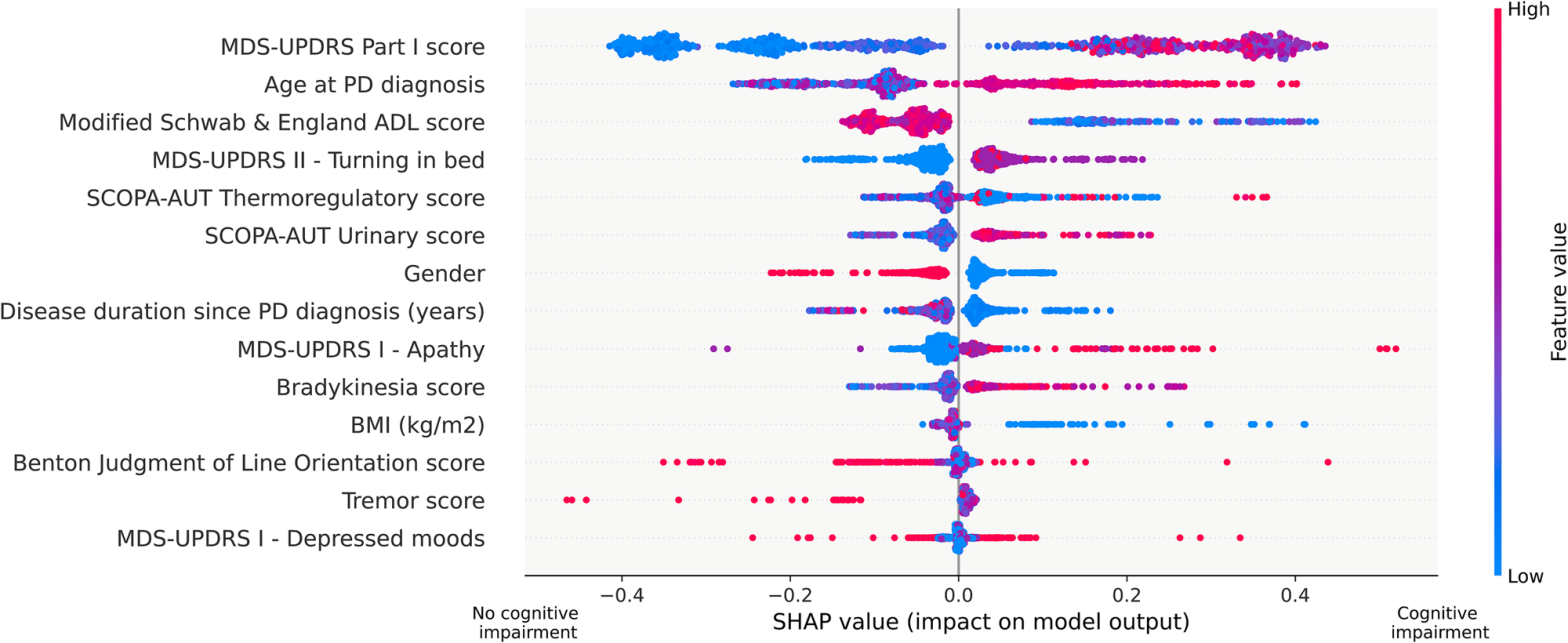
SHAP value plot revealing key predictors’ influence on *SCD* classification in the cross-cohort analysis. Each row shows a predictor’s impact on subjective cognitive decline (*SCD*) classification, with SHAP values indicating the direction and magnitude of effect. Points represent individual patients, with colors indicating the predictor’s value (red = high, blue = low). Positive SHAP values (right side) indicate increased likelihood of *SCD*, while negative values (left side) suggest decreased likelihood. The MDS-UPDRS Part I score shows the strongest effect, with higher scores (red) associated with increased *SCD* risk. Age at PD diagnosis demonstrates the second strongest impact, with later onset (red) correlating with higher *SCD* probability.

For *SCD*, age at PD diagnosis and Benton JLO ranked highly (Fig. 2 and Supplementary Fig. 10), with age at PD diagnosis showing negative correlations with MoCA and Benton JLO (Supplementary Table 12).

**Fig. 3:**
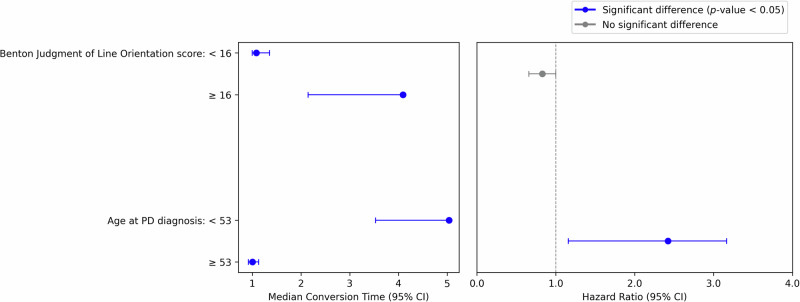
Forest plot of median conversion times (years) and hazard ratios for key predictors of time-to-*PD-MCI* in the cross-cohort analysis. Forest plot showing the relationship between two key predictors (Benton Judgment of Line Orientation score and age at PD diagnosis) and the development of mild cognitive impairment (*PD-MCI*). The left panel displays median conversion times with 95% confidence intervals (CIs), stratified by predictor thresholds (≥16 vs <16 for Benton score; ≥53 vs <53 years for age at PD diagnosis). Solid blue lines indicate statistically significant differences between groups (*p*-value < 0.05), while grey lines indicate non-significant differences. The right panel shows corresponding hazard ratios (HR) with 95% CIs, where HR greater than 1 indicate increased risk of *PD-MCI* for the higher category compared to the reference group (lower category). For age at PD diagnosis, patients diagnosed at ≥53 years show significantly higher risk of developing *PD-MCI* compared to those diagnosed earlier.

**Fig. 4:**
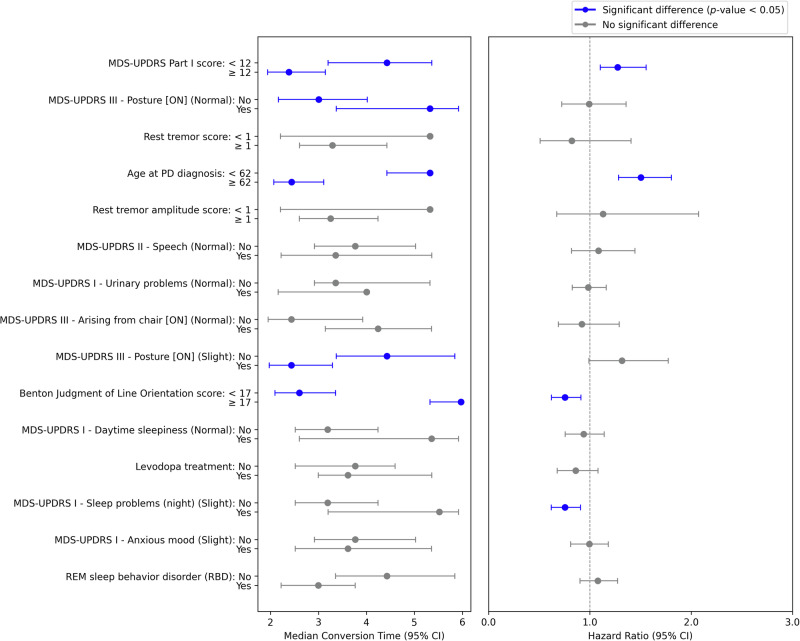
Forest plot of time-to-*SCD* predictors showing median conversion times (years) and hazard ratios in the cross-cohort analysis. The plot illustrates how multiple clinical predictors affect subjective cognitive decline (*SCD*). The left panel shows median time to *SCD* onset with 95% confidence intervals (CIs), comparing subgroups for each predictor. Solid blue lines indicate statistically significant differences between groups (*p*-value < 0.05), while grey lines indicate non-significant differences. Key predictors include MDS-UPDRS Part I score (≥12 vs <12), postural abnormalities, tremor characteristics, and age at PD diagnosis (≥62 vs <62 years). The right panel displays corresponding hazard ratios (HR) with 95% CIs, where values > 1 indicate increased risk. Notable findings include a significantly higher *SCD* risk for patients diagnosed after age 62 and those with higher MDS-UPDRS Part I scores. Some features such as sleep problems and REM sleep behavior disorder show wider confidence intervals, suggesting more uncertainty in their predictive value.

In time-to-*PD-MCI* analysis, patients diagnosed at age 53 or older had a nearly 2.4-fold higher risk of CI compared to those at a younger age (Fig. 3). In the time-to-*SCD* analysis, patients diagnosed at age 62 or older had a 1.5-fold higher risk (Fig. 4). Factors such as MDS-UPDRS Part I, disease duration, tremors, and male sex were associated with increased *SCD* risk (Fig. 2). Meanwhile, lower Modified Schwab & England Activities of Daily Living (ADL) scores correlated with perceived CI.

Non-motor symptoms (MDS-UPDRS Part I, SCOPA-AUT) including sleep disturbances were associated with increased *SCD* risk, highlighting CI’s multifactorial nature. BMI was positively associated with *PD-MCI* (not *SCD*), and thermoregulatory and sexual dysfunction correlating with *SCD* alone (Supplementary Table 13).

Overall, cognitive decline in PD is associated with multiple clinical features, with distinct patterns for objective CI and subjective patient reports, highlighting the need for comprehensive, multifactorial approaches for prediction and clinical management.

### Decision curve and calibration analysis

Decision curve analysis (DCA) and calibration analysis were performed to assess model reliability and clinical utility for *PD-MCI* and *SCD* outcomes. For *PD-MCI* classification, the AdaBoost-optimized model showed a high area under the net benefit curve (AUNBC) (0.23) and a calibration slope of 1.13 (Supplementary Fig. 11 and Supplementary Table 14), indicating high model reliability. In time-to-*PD-MCI* analysis, the penalized Cox-optimized model also achieved a high AUNBC (0.22; Supplementary Fig. 12), with a calibration slope of 1.30, showing slight overestimation for higher risks but overall reasonable agreement.

For *SCD* classification, the FIGS model provided the best calibration slope (0.74), though other models achieved higher AUNBC (0.08; Supplementary Fig. 13). Time-to-*SCD* analysis faced calibration challenges despite reasonable AUNBC (0.10; Supplementary Fig. 14) but lower calibration slopes, suggesting good risk distinction but less accuracy in estimating *SCD* risk.

These results highlight that model performance, net benefit and calibration need to be considered separately. While *PD-MCI* models show promise for clinical decision-making, *SCD* models, particularly for time-to-event prediction, need refinement for better calibration and clinical reliability.

## Discussion

When assessing cognition in PD patients, both objective and subjective measures should be considered. Our study revealed a limited correlation between *PD-MCI* and *SCD* (0.41 for classification and 0.49 for time-to-event analysis in uncensored data), indicating that, while these outcomes share common predictors, they also capture distinct aspects of CI. Notably, the median time-to-*PD-MCI* and time-to-*SCD* (uncensored data) from the cross-cohort analysis were 1.84 and 3.01 years, respectively, highlighting differences in CI timing between objective and subjective measures.

This study used a multi-cohort approach to identify consistent predictors of *PD-MCI* and *SCD* in PD, ensuring broader model applicability by integrating diverse cohort data. Key strengths of this approach are that it mitigates cohort-specific biases^26^ of single-cohort studies and evaluates model performance more robustly and reliably across heterogeneous populations, thereby supporting the identification of predictors that are consistent and transferable across settings. While further model improvements will be needed for future clinical translation, this increases the utility of the findings for real-world applications across distinct patient populations, where patient characteristics, assessment protocols, and healthcare settings often vary.

Differences in predictive performance across cohorts highlight variations in clinical characteristics and data distributions. The LuxPARK cohort had an older average age at PD diagnosis and longer disease duration, ICEBERG participants had significantly lower average body weight and BMI, and the PPMI cohort exhibited milder disease severity with lower MDS-UPDRS and SCOPA-AUT scores. These baseline differences highlight the need to consider cohort-specific factors when interpreting model performance and the challenges of developing universally applicable predictive tools for CI in PD.

Cross-cohort models showed greater performance robustness than single-cohort models, maintaining predictive power without sacrificing accuracy in more heterogeneous datasets. Model stability is an important aspect of ML studies that is often underreported. Many previous studies have focused solely on average predictive performance, which is insufficient for comprehensive model evaluation. High variability can limit the reproducibility and generalizability of findings. Our empirical analyses confirm that integrating data from multiple cohorts improves model stability in terms of the variance of performance estimates across the CV cycles, while maintaining competitive average performance estimates compared to less reliable single-cohort models, an important step toward developing trustworthy tools for clinical use. Training on multiple cohorts also enabled the models to capture broader patient characteristics, increasing their generalizability. Conversely, single-cohort models were more affected by cohort-specific biases and smaller sample sizes, limiting their broader applicability.

Our study also identified stable predictors of CI that persist across different clinical settings and patient populations. While age at PD diagnosis was the top-ranked predictor in both single- and multi-cohort settings, other features, such as the Benton JLO, gained importance in the cross-cohort models, replacing less consistently selected features such as the MoCA that dominated the single-cohort results. This shift highlights the added value of multi-cohort integration in identifying robust features less influenced by cohort-specific characteristics and more likely to generalize across populations. These findings support the use of cross-cohort modeling to identify consistent predictors that could ultimately inform early intervention strategies.

Additionally, the cross-cohort validation framework ensures a rigorous performance assessment, by testing models across populations with different demographic and clinical characteristics. Comparable performance across cohorts with varying disease severity, age distributions, and assessment protocols suggests that the identified predictors are robust and clinically meaningful across different healthcare settings. These findings reflect a significant advancement toward the implementation of clinically useful, explainable artificial intelligence (XAI) tools that can function across healthcare systems.

The increased robustness of cross-cohort models and their stable significant performance demonstrated in our study builds upon previous research in CI prediction. Earlier studies relied on single-cohort data, limiting generalizability due to cohort-specific biases. Some of these studies reported higher AUCs, but they were not validated in distinct cohorts (e.g., 0.71 for a two-year longitudinal study^2^ and 0.80 with APOE status inclusion^34^). In cognitive assessment tools, MoCA demonstrated stronger predictive power for *PD-MCI* (AUC 0.83) than Mini-Mental State Examination (MMSE) (AUC 0.67)^35^, while studies incorporating biological factors and deep radiomic features reported AUCs ranging from 0.61 for *PD-MCI*^36^ to 0.89 and 0.81 for objective and subjective CI, respectively^37^. However, these performance estimates may be optimistic, as they do not account for cross-study variability and lack external validation. Our cross-cohort approach achieved hold-out AUC/C-index values of 0.67 for *PD-MCI* classification, 0.65 for time-to-*PD-MCI*, 0.72 for both *SCD* classification and for time-to-*SCD*. While these values are lower compared to the best single-cohort study results, they reflect a more thorough cross-cohort assessment and more robust models that account for real-world heterogeneity and reduce overfitting risks. This highlights an important limitation of the existing literature, strong average within-cohort performance does not guarantee model generalizability or robustness. By integrating data from three independent cohorts with varying characteristics and follow-up structures, our models reduce the sampling bias. Despite the added complexity and challenges of multi-cohort modeling, this approach yields more stable and reproducible predictors^26^, representing an essential step toward translating CI research findings into clinical decision support tools.

The DCA highlighted the potential utility of cross-cohort models to guide future intervention studies, showing higher net benefit across a broad range of threshold probabilities. We note that the interpretation of the net benefit depends on the assumed impact of possible early interventions. While the magnitude of the effect of a hypothetical treatment does not change the net benefit analysis, the practical utility of the model scales with the assumed impact of potential interventions. Calibration analysis further confirmed that the cross-cohort model for *PD-MCI* classification and time-to-*SCD* exhibited a high calibration slope, supporting its reliability for risk estimation in external applications.

In our feature ranking analyses, age at PD diagnosis emerged as a key predictor of CI, with older patients at higher risk, aligning with previous findings on late-onset PD^38^, where patients diagnosed at age ≥53 years had a nearly 2.4-fold increased risk of developing CI. This association may reflect the increased vulnerability of age-related brain networks to neurodegenerative processes^39^. While both early- and late-onset PD exhibit altered functional connectivity of brain networks^40^, late-onset patients may experience faster cognitive decline. These findings highlight both challenges and opportunities for clinical application, emphasizing the need for timely intervention, particularly for high-risk patients.

Sex differences were observed in *SCD*, with men more likely to report CI. Women generally performed better on cognitive tests^41,42^, and had lower reported CI scores, suggesting sex-specific aspects of CI^43^. While the increased risk of *PD-MCI* in men with PD is well-documented^41,42^ and our cross-cohort analysis indicated sex as an informative predictor variable for *SCD*, a significant association was not detected in the single-cohort or *PD-MCI* analyses included in this study. This suggests that sex-related differences may influence self-perceived cognitive decline more prominently than objectively measured impairment.

Visuospatial deficits, as measured by the Benton JLO test, emerged as an important predictor, consistent with previous research^4,24,31^. These impairments may manifest as challenges in judging distances or mentally rotating objects, and are commonly evaluated using tasks such as the clock-drawing test^44^. Notably, women achieved higher global cognition scores, whereas men performed better on visuospatial tasks^45,46^, which may reflect biological and psychosocial factors. Hormonal differences may contribute to these variations, highlighting the importance of considering sex-specific factors in cognitive assessment and intervention^47,48^.

Non-motor symptoms, particularly autonomic dysfunction as measured by the SCOPA-AUT, were strongly associated with *SCD*, emphasizing the multifactorial nature of CI in PD^49^. In particular, gastrointestinal tract symptoms, such as constipation, have been linked to CI^50,51^, and autonomic dysfunction has been associated with PD progression^52^. Additionally, sleep disturbances may also influence CI, as poor sleep quality is known to exacerbate cognitive difficulties, including impaired memory processing^53^. Our analyses found that sleep problems at night, assessed via the MDS-UPDRS Part I, showed a significant hazard ratio (HR) in the time-to-*SCD* model. Patients with sleep disorders often experience challenges with attention, memory, and problem-solving^54^. These associations may reflect shared underlying mechanisms, such as neurotransmitter dysregulation^55,56^, that affect multiple functional domains simultaneously, rather than direct causal links. Therefore, it is important to interpret such predictors within a multivariate modeling framework to account for potential variable interactions.

ML models allowed us to explore the predictive features in greater depth than traditional statistical approaches, particularly in a multivariate context. Unlike univariate analyses, ML models can consider the interdependencies between variables, facilitating the distinction between direct and indirect causal variable relationships and non-causal associations. By applying ML techniques across multiple cohorts, we identified robust predictors that hold promise for precision medicine and general clinical practice. The identification of consistent predictors across cohorts provides clinicians with valuable tools for the early identification of high-risk patients, facilitating timely interventions and personalized management strategies. Given the reliance on commonly collected clinical variables, optimized versions of the developed models could be integrated into digital health platforms, including telemedicine systems or mobile health applications, for remote cognitive screening or ongoing monitoring. This integration into digital tools offers a scalable path toward precision medicine in PD and could also contribute to the broader translation of improved multi-modal ML models into practical clinical applications. However, important limitations remain to be addressed. Generalizability may be restricted by the populations covered in the cohorts, and the exclusion of variables unavailable across all cohorts. Differences in predictive performance may also arise from varying sample sizes and patient characteristics. Additionally, while our models showed significant predictive performance in a challenging cross-study setting, they are not yet directly applicable for clinical use, and further optimization and validation in prospective studies are needed before clinical translation. Despite these challenges, cross-cohort ML approaches provide a robust basis to further extend and optimize CI prediction models towards clinically relevant, digitally deployable tools for precision medicine applications in PD. Future research should further expand, optimize, and validate these predictors in diverse populations and explore their application to guide early intervention studies.

## Methods

### Inclusion criteria and sample characteristics

This study used data from three PD cohorts (Table 2): LuxPARK (number of subjects: 467 *PD-MCI*+, 64 *PD-MCI−*; 279 *SCD*+, 133 *SCD–*)^27^, a longitudinal monocentric observational study in Luxembourg and the surrounding Greater Region; PPMI (393 *PD-MCI*+, 232 *PD-MCI−*; 147 *SCD*+, 377 *SCD−*)^28^, a multicenter observational study; and ICEBERG (56 *PD-MCI*+, 61 *PD-MCI−*; 61 *SCD*+, 56 *SCD−*)^29^, a French early-stage PD cohort (see detailed cohort descriptions in the Supplementary Material). Participants met two criteria:

- A PD diagnosis according to the UK Parkinson’s Disease Society Brain Bank (UKPDSBB) criteria^57^ for the LuxPARK and ICEBERG, or, for the PPMI, the presence of at least two of the following: resting tremor, bradykinesia, or rigidity (with resting tremor or bradykinesia required)^58^.
- The clinically confirmed presence or absence of *PD-MCI* or *SCD* within four years of the baseline visit.

**Table 2:** Inclusion criteria and occurrence of mild cognitive impairment (*PD-MCI*) and subjective cognitive decline (*SCD*)

	Mild cognitive impairment (*PD-MCI*)				Subjective cognitive decline (*SCD*)			
Cohort	Inclusion criteria (1)	Inclusion criteria (2)	Events (*PD-MCI* Classification)	Events (Time-to-*PD-MCI*)	Inclusion criteria (1)	Inclusion criteria (2)	Events (*SCD* classification)	Events (time-to-*SCD*)
LuxPARK	706	531	467 (87.9%)	471 (88.7%)	706	412	279 (67.7%)	291 (70.6%)
PPMI	1624	625	393 (62.9%)	462 (74.2%)	1624	524	147 (28.1%)	285 (54.4%)
ICEBERG	162	117	56 (47.9%)	56 (47.9%)	162	117	61 (52.1%)	61 (52.1%)
Total	2492	1273	916 (72.0%)	989 (77.7%)	2492	1053	487 (46.2%)	637 (60.5%)

The numbers of PD patients who met inclusion criteria, and the occurrence of mild cognitive impairment (*PD-MCI*) and subjective cognitive decline (*SCD*) among these patients considered in the 4-year classification analysis and the time-to-event analyses for the LuxPARK, PPMI, and ICEBERG cohorts.

All participants enrolled in the Luxembourg Parkinson’s Study, the ICEBERG cohort, and the PPMI cohort provided written informed consent. The individual studies received approval from the National Research Ethics Committee (CNER Ref: 201407/13) for the Luxembourg Parkinson’s Study, IRB Paris VI (RCB: 2014-A00725-42) for the ICEBERG cohort, and from multiple institutional review boards/ethics committees at all participating sites for PPMI. All studies adhered to the principles outlined in the Declaration of Helsinki. Additionally, the Luxembourg Parkinson’s Study, ICEBERG, and PPMI are registered with ClinicalTrials.gov under the identifiers NCT05266872, NCT02305147, and NCT04477785, respectively.

The *PD-MCI* status was defined as positive (*PD-MCI*+) for a MoCA score <26, and as negative otherwise (*PD-MCI-*)^3^, while the *SCD* status was defined as positive (*SCD*+) if the score for the MDS-UPDRS Part I item 1.1 was above 1, and negative (*SCD−*) otherwise^59^. Level I MDS criteria were used for uniform cognitive profiling. Single-cohort and multi-cohort analyses followed a consistent workflow, detailed in the Supplementary Material.

Clinical characteristics were assessed for *PD-MCI* and *SCD* classification within four years of the baseline clinical visit, with time-to-event analysis tracking conversion from *PD-MCI*-/*SCD-* to *PD-MCI*+/*SCD+* or the end of follow-up if censored.

### Machine learning analysis of cognitive impairment

We developed a comprehensive ML framework to evaluate predictors of CI in PD, including data preprocessing, model training, and validation for classification and time-to-event analyses.

Prior to analysis, data preprocessing was performed to ensure the comparability of the relevant cohort variables. This included variable aggregation (Supplementary Table 15), missing value imputation for baseline features^60,61^, cross-study normalization (mean centering^62^, standardization, quantile normalization^63,64^, ComBat^65,66^, Ratio-A^67^, and M-ComBat^68^), undersampling^69^, and feature selection^70^. Feature selection included Recursive Feature Elimination (RFE) and Bidirectional Stepwise Feature Selection, both assessed via CV (for details on all data preprocessing and CV steps, see section “Data preprocessing” and “Cross-validation” in the Supplementary Material).

Model performance was evaluated using a two-level nested CV^71^. Firstly, the data were split into a training (67%) and a test set (33%), using stratification by cohort to maintain the distribution of cohorts. Within the training set, 5-fold stratified cross-validation was used to provide a first estimate of the average performance of the model and its variability. In addition, to assess generalizability across diverse populations, a leave-one-cohort-out validation strategy was applied. This involved training models on two cohorts and testing them on the third, enabling the evaluation of model robustness across cohorts with different demographics, disease characteristics, and follow-up durations. Hyperparameter tuning and feature selection were performed within the nested CV loops to minimize overfitting and ensure fair model evaluation (see Supplementary Figs. 15–17 and “*Model optimization and evaluation”* below).

For machine learning, nine algorithms were used for CI classification (*PD-MCI*+ or *SCD*+): AdaBoost^72,73^, CART^74^, CatBoost^75^, C4.5 trees^76^, FIGS^77^, GOSDT-GUESSES^78^, Gradient Boosting (GBoost)^79^, Hierarchical Shrinkage (HS)^80^, and XGBoost^81^. For time-to-event analysis, eight approaches were applied: Component-wise Gradient Boosting (CW-GBoost)^82^, Survival Trees^83^, Extra Survival Trees^84^, Survival GBoost^70^, Linear Support Vector Machine (LSVM), Naive Linear Support Vector Machine (NLSVM)^85^, Penalized Cox regression^86,87^, and Survival Random Forests (RF)^88^.

Hyperparameters were optimized using a nested CV to maximize the average area under the curve (AUC; classification) and concordance index (C-index; time-to-event). Single-cohort models were trained and validated within individual cohorts, while multi-cohort approaches included cross-cohort analyses (training on part of the samples from all cohorts, and testing on independent hold-out samples from all cohorts) and leave-one-cohort-out analyses (training on two cohorts and testing on the third cohort, see the supplementary material). As undersampling was applied to the training sets to address class imbalances, this nested CV structure inherently performs undersampling 15 times across different data partitions (5 outer folds × 3 inner folds), providing sufficient repeated sampling to mitigate potential sampling bias.

### Interpretation of models and predictors

We applied SHAP value analysis^89^ to interpret prediction models and assess feature importance. The log-rank test compared Kaplan-Meier (KM) curves to assess time-to-*PD-MCI*/*SCD* differences across subgroups.

SHAP-derived HR were used to quantify the relative risk of *PD-MCI*/*SCD*, linking predictors to outcomes. Bootstrapped 95% confidence intervals (CIs) were computed to assess uncertainty around each HR estimate^90^.

### Evaluation of model performance and stability

Model performance and stability were assessed using the CV-AUC and CV-C for *PD-MCI*/*SCD* prediction. 95% CIs were estimated via bootstrapping with 1000 resamples, using the 2.5^th^ and 97.5^th^ percentiles as CIs bounds. The effect of cross-study normalization on the hold-out test set performance was evaluated using DeLong’s test for classification^91^ and the one-shot non-parametric test for time-to-event analysis^92^. *P*-values were adjusted for multiple comparisons^93^ and Bayesian signed-rank tests used to assess model performance across cohorts^94^. Model stability was evaluated by computing the standard deviation of performance statistics across CV cycles.

### Predictor selection statistics across cohorts

We used a common data model to identify informative baseline predictors across cohorts. Feature selection statistics were compared across single cohort analyses, calculating selection frequency over the CV cycles. Focusing on models with the highest CV-AUC/CV-C, features derived from the same categorical variable (via one-hot encoding) were grouped to avoid inflated importance. This approach ensured consistent and robust predictor identification across clinical settings.

### Statistical analysis

We assessed group differences, distributions, and relationships between variables. To compare baseline characteristics between groups, the Mann–Whitney U test was used for non-normally distributed variables, the two-sample t-test for normally distributed variables, and Fisher’s exact test for categorical variables. The normality assumption was checked using the Shapiro–Wilk test to choose between parametric and non-parametric tests.

Cohort comparisons used ANOVA with Tukey’s HSD for normal distributions and Kruskal–Wallis with Dunn’s test for non-normal distributions. Variable associations were assessed using Spearman’s correlation for continuous/ordinal variables, the point-biserial correlation for binary and continuous/ordinal variables, Matthew’s Correlation Coefficient (MCC) for binary variables, and Kendall’s tau for ordinal variables. Significance was defined at *p* < 0.05 across all tests. As a final statistic, the median time to 50% *PD-MCI/SCD* conversion was examined to provide a clinically relevant measure of CI progression.

### Assessment of clinical utility: decision curve and calibration analysis

Decision curve and calibration analysis were used to assess the models’ clinical utility and reliability:

DCA was applied to the hold-out test set to assess the net benefit over “treating all” or “none” scenarios^95^. The AUNBC was used to quantify clinical utility, with a larger AUNBC signifying a greater decision advantage. To evaluate the significance of AUNBC differences, bootstrapped hypothesis testing (1000 replicates) was used^96^.

For the Calibration analysis, the agreement between predicted probabilities and observed outcomes was assessed^97^. For time-to-*PD-MCI*/*SCD*, 4-year predicted probabilities were compared with KM estimates^98^, determining the calibration slope and mean square error (MSE).

**Fig. 5:**
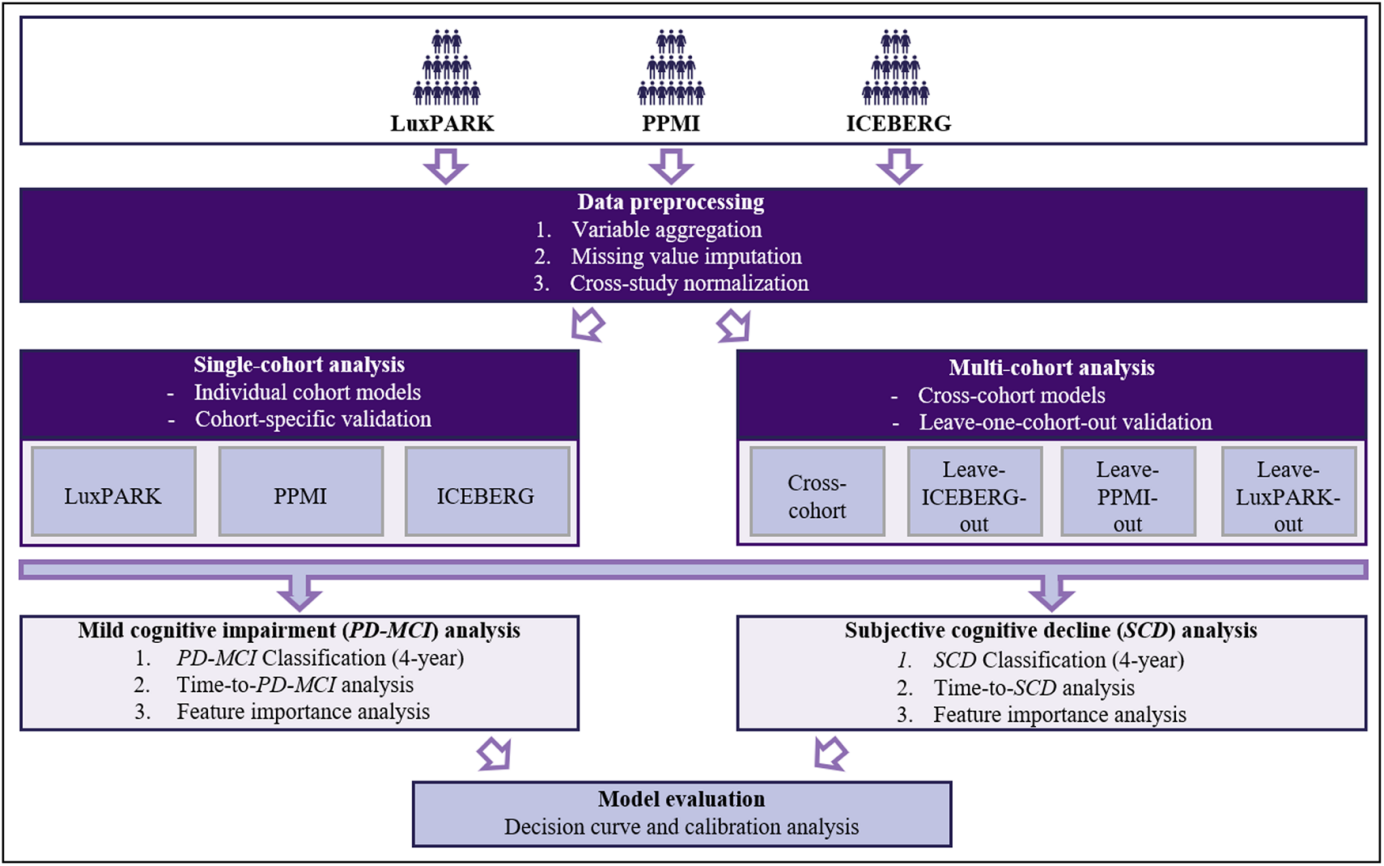
Machine learning pipeline for predicting cognitive impairment in Parkinson’s disease. Machine learning analysis pipeline for predicting cognitive impairment in Parkinson’s Disease. Schematic representation of the data processing and analysis workflow. Input data from three independent cohorts (LuxPARK, PPMI, and ICEBERG) is pre-processed and then analyzed using both single-cohort and multi-cohort approaches. These analyses are applied to predict both mild cognitive impairment (*PD-MCI*) and subjective cognitive decline (*SCD*) outcomes in Parkinson’s disease. The models are evaluated using cross-validation, decision curve and calibration analyses.

Normalization and statistical analyses were performed using the R statistical programming language (v4.2.1). Python-3.8.6-GCCcore-10.2.0 was used for data processing and ML analyses. Figure 5 provides an overview of the workflow.

## Supplementary information

Supplementary information

## Data Availability

The LuxPARK clinical dataset used in this study was obtained from the National Centre of Excellence in Research on Parkinson’s Disease (NCER-PD). The dataset for this manuscript is not publicly available as it is linked to the Luxembourg Parkinson’s Study and its internal regulations. Any requests for accessing the dataset can be directed to request.ncer-pd@uni.lu. Further data used in the preparation of this article were obtained on May 9, 2024 from the Parkinson’s Progression Markers Initiative (PPMI) database (www.ppmi-info.org/data, RRID:SCR 006431). For up-to-date information on the study, please visit the PPMI website (www.ppmiinfo.org). Data from the ICEBERG cohort analyzed during this study is available from the corresponding study group (jean-christophe.corvol@aphp.fr, marie.vidailhet@aphp.fr).

## Code availability

The open-source code is accessible in the following GitLab repository under the MIT license: https://gitlab.com/uniluxembourg/lcsb/bds/ml-cognitive-impairment.
