# Supplementary material for: Multi-cohort machine learning identifies predictors of cognitive impairment in Parkinson’s disease
Source: NPJ Digit Med. 2025 Jul 26;8:482. doi: 10.1038/s41746-025-01862-1 (PMC12297691; doi:10.1038/s41746-025-01862-1)
Supplement: Supplementary file 1 — Supplementary information [file 41746_2025_1862_MOESM1_ESM.pdf]

# Multi-cohort Machine Learning Identifies Predictors of Cognitive Impairment in Parkinson's Disease

Rebecca Ting Jiin Loo, PhD[1], Lukas Pavelka, MD[2], Graziella Mangone, MD, PhD[3], Fouad Khoury, MD, PhD[3], Marie Vidailhet, MD, PhD[3], Jean-Christophe Corvol, MD, PhD[3], Enrico Glaab, PhD[1↑] on behalf of the NCER-PD Consortium

[1]*Biomedical Data Science Group, Luxembourg Centre for Systems Biomedicine (LCSB), University of Luxembourg, Esch-sur-Alzette, Luxembourg*
[2]*Transversal Translational Medicine, Luxembourg Institute of Health (LIH), Strassen, Luxembourg*
[3]*Sorbonne Université, Paris Brain Institute - ICM, Inserm, CNRS, Assistance Publique Hôpitaux de Paris, Pitié-Salpêtrière Hospital, Department of Neurology, Paris 75013, France*
[↑]*Contact: enrico.glaab@uni.lu*

# Supplementary materials

Model performance statistics for mild cognitive impairment (PD-MCI) and subjective cognitive decline (SCD) across different cohorts ..... 1
Stability of the predictive models for predicting the occurrence of cognitive impairment ..... 11
Model performance statistics for cognitive impairment prognosis: Unnormalized vs. normalized models compared across multiple cohorts ..... 13
Comparative baseline clinical feature analysis across cohorts ..... 15
SHAP value for the cross-cohort analysis ..... 16
Correlation analysis of predictors ..... 17
Evaluation of predictive models using decision curve and calibration analysis ..... 19
Cohorts ..... 22
Methods ..... 23
    Data preprocessing ..... 23
    Cross-validation ..... 26
        Machine learning classification ..... 28
        Time-to-event analysis ..... 29
References ..... 29

# Model performance statistics for mild cognitive impairment (*PD-MCI*) and subjective cognitive decline (*SCD*) across different cohorts

**Supplementary Table 1**: **Cross-validated and hold-out AUCs for *PD-MCI* classification in single-cohort analyses.** Summary of predictive performance metrics for mild cognitive impairment (*PD-MCI*) classification for the single-cohort analyses, considering each cohort (LuxPARK, PPMI, and ICEBERG) independently. The table presents cross-validated and hold-out AUC values and the number of features used in each model. Models with the highest average cross-validated AUC scores in each cohort analysis are highlighted in bold. The model with the highest hold-out AUC score is indicated in *italics*. The "number of features" column includes the number of candidate features selected during cross-validation (shown in brackets) and the subset of features demonstrating significant predictive impact identified through permutation importance analysis (preceding the brackets).

| **Single-cohort analyses** | | | | | | | | | |
|---|---|---|---|---|---|---|---|---|---|
| **Algorithm** | **LuxPARK** | | | **PPMI** | | | **ICEBERG** | | |
| | **Mean (95% CIs)** | **Hold-out AUC** | **Number of features** | **Mean (95% CIs)** | **Hold-out AUC** | **Number of features** | **Mean (95% CIs)** | **Hold-out AUC** | **Number of features** |
| AdaBoost | **0.701 (0.587, 0.768)** | **0.557** | **3 (9)** | 0.697 (0.682, 0.711) | 0.663 | 1 (1) | **0.600 (0.519, 0.692)** | **0.451** | **6 (11)** |
| CART | 0.680 (0.611, 0.749) | 0.503 | 8 (14) | 0.686 (0.661, 0.710) | 0.663 | 1 (1) | 0.550 (0.514, 0.585) | 0.500 | 1 (1) |
| CatBoost | 0.699 (0.625, 0.775) | 0.502 | 10 (15) | 0.693 (0.647, 0.734) | 0.637 | 7 (12) | ***0.581 (0.496, 0.674)*** | ***0.674*** | ***5 (7)*** |
| C4.5 | 0.637 (0.565, 0.701) | 0.456 | 4 (6) | **0.701 (0.668, 0.733)** | **0.694** | **2 (3)** | 0.534 (0.429, 0.656) | 0.527 | 4 (8) |
| FIGS | 0.654 (0.616, 0.702) | 0.530 | 3 (7) | 0.686 (0.661, 0.710) | 0.663 | 1 (1) | 0.549 (0.482, 0.616) | 0.511 | 3 (6) |
| GOSDT-GUESSES | 0.634 (0.575, 0.695) | 0.534 | 9 (18) | 0.617 (0.574, 0.659) | 0.659 | 21 (38) | 0.503 (0.430, 0.601) | 0.563 | 6 (13) |
| GBoost | 0.657 (0.574, 0.720) | 0.589 | 8 (17) | ***0.688 (0.666, 0.714)*** | ***0.707*** | ***5 (20)*** | 0.510 (0.416, 0.604) | 0.479 | 4 (8) |
| HS | 0.672 (0.574, 0.732) | 0.565 | 3 (6) | 0.686 (0.661, 0.710) | 0.663 | 1 (1) | 0.515 (0.452, 0.577) | 0.500 | 1 (4) |
| XGBoost | ***0.697 (0.646, 0.757)*** | ***0.702*** | ***5 (6)*** | 0.653 (0.585, 0.710) | 0.645 | 32 (50) | 0.597 (0.467, 0.718) | 0.534 | 18 (25) |

**Supplementary Table 2. Predictive performance metrics for time-to-*PD-MCI* in single-cohort analyses.** Summary of predictive performance metrics for time to mild cognitive impairment (*PD-MCI*) for the single cohort analyses. The table presents cross-validated and hold-out C-indices and the number of features used in each model. Models with the highest average cross-validated C-indices in each cohort analysis are highlighted in bold. The model with the highest hold-out C-index is indicated in *italics*. The "number of features" column includes the number of candidate features selected during cross-validation (shown in brackets) and the subset of features demonstrating significant predictive impact identified through permutation importance analysis (preceding the brackets).

| **Single-cohort analyses** | | | | | | | | | |
|---|---|---|---|---|---|---|---|---|---|
| | **LuxPARK** | | | **PPMI** | | | **ICEBERG** | | |
| **Algorithm** | **Mean (95% CIs)** | **Hold-out C-index** | **Number of features** | **Mean (95% CIs)** | **Hold-out C-index** | **Number of features** | **Mean (95% CIs)** | **Hold-out C-index** | **Number of features** |
| CW-GBoost | **0.720 (0.675, 0.762)** | **0.633** | **10 (24)** | 0.612 (0.598, 0.626) | 0.601 | 10 (16) | 0.570 (0.448, 0.711) | 0.558 | 5 (8) |
| Extra Survival | ***0.676 (0.625, 0.725)*** | ***0.634*** | ***94 (132)*** | 0.657 (0.617, 0.691) | 0.641 | 13 (13) | **0.644 (0.525, 0.807)** | **0.542** | **45 (77)** |
| Survival GBoost | 0.640 (0.573, 0.702) | 0.589 | 8 (13) | 0.660 (0.632, 0.687) | 0.682 | 14 (23) | 0.521 (0.471, 0.571) | 0.577 | 10 (21) |
| LSVM | 0.630 (0.579, 0.666) | 0.548 | 19 (19) | ***0.646 (0.591, 0.693)*** | ***0.722*** | ***28 (28)*** | 0.625 (0.539, 0.711) | 0.591 | 11 (11) |
| NLSVM | 0.606 (0.564, 0.644) | 0.611 | 174 (174) | 0.627 (0.591, 0.662) | 0.682 | 36 (36) | 0.564 (0.506, 0.629) | 0.521 | 77 (79) |
| Penalized Cox | 0.652 (0.596, 0.708) | 0.597 | 3 (3) | 0.631 (0.596, 0.665) | 0.677 | 1 (3) | ***0.500 (0.466, 0.535)*** | ***0.614*** | ***7 (7)*** |
| Survival RF | 0.694 (0.658, 0.750) | 0.590 | 4 (4) | **0.660 (0.600, 0.707)** | **0.644** | **9 (11)** | 0.603 (0.493, 0.696) | 0.495 | 62 (80) |
| Survival Trees | 0.657 (0.612, 0.699) | 0.611 | 14 (24) | 0.626 (0.584, 0.656) | 0.658 | 5 (11) | 0.541 (0.446, 0.656) | 0.526 | 4 (5) |

**Supplementary Table 3. Predictive performance metrics for *SCD* classification in single-cohort analyses.** Summary of predictive performance statistics for subjective cognitive decline (*SCD*) classification. The table presents cross-validated and hold-out AUC values for single-cohort analyses and the number of features used in each model. Models with the highest average cross-validated AUC scores in each cohort analysis are highlighted in bold. The model with the highest hold-out AUC score is indicated in *italics*. The "number of features" column includes the number of candidate features selected during cross-validation (shown in brackets) and the subset of features demonstrating significant predictive impact identified through permutation importance analysis (preceding the brackets).

| **Single-cohort analyses** | | | | | | | | | |
|---|---|---|---|---|---|---|---|---|---|
| **Algorithm** | **LuxPARK** | | | **PPMI** | | | **ICEBERG** | | |
| | **Mean (95% CIs)** | **Hold-out AUC** | **Number of features** | **Mean (95% CIs)** | **Hold-out AUC** | **Number of features** | **Mean (95% CIs)** | **Hold-out AUC** | **Number of features** |
| AdaBoost | ***0.643 (0.574, 0.717)*** | ***0.631*** | ***7 (19)*** | 0.665 (0.598, 0.718) | 0.599 | 4 (5) | ***0.653 (0.427, 0.900)*** | ***0.615*** | ***1 (1)*** |
| CART | 0.632 (0.569, 0.679) | 0.599 | 7 (14) | 0.638 (0.615, 0.665) | 0.570 | 1 (5) | 0.677 (0.613, 0.733) | 0.538 | 6 (7) |
| CatBoost | 0.693 (0.648, 0.738) | 0.604 | 13 (28) | **0.709 (0.638, 0.764)** | **0.663** | **8 (16)** | 0.703 (0.590, 0.817) | 0.596 | 7 (14) |
| C4.5 | 0.668 (0.647, 0.685) | 0.543 | 5 (15) | 0.639 (0.613, 0.666) | 0.570 | 1 (6) | ***0.628 (0.503, 0.775)*** | ***0.615*** | ***2 (3)*** |
| FIGS | 0.667 (0.588, 0.730) | 0.518 | 8 (17) | 0.649 (0.617, 0.681) | 0.570 | 1 (3) | ***0.643 (0.517, 0.770)*** | ***0.615*** | ***2 (4)*** |
| GOSDT-GUESSES | 0.616 (0.574, 0.668) | 0.570 | 19 (30) | 0.619 (0.547, 0.698) | 0.606 | 14 (27) | **0.732 (0.587, 0.875)** | **0.519** | **7 (7)** |
| GBoost | 0.624 (0.559, 0.674) | 0.580 | 15 (26) | 0.656 (0.628, 0.685) | 0.649 | 15 (23) | 0.673 (0.507, 0.850) | 0.500 | 3 (5) |
| HS | 0.625 (0.565, 0.680) | 0.482 | 1 (2) | 0.649 (0.617, 0.681) | 0.570 | 1 (3) | ***0.643 (0.517, 0.770)*** | ***0.615*** | ***2 (4)*** |
| XGBoost | **0.694 (0.596, 0.767)** | **0.592** | **13 (15)** | ***0.676 (0.640, 0.706)*** | ***0.698*** | ***27 (35)*** | 0.578 (0.478, 0.692) | 0.500 | 19 (24) |

**Supplementary Table 4. Predictive performance metrics for time-to-*SCD* in single-cohort analyses.** Summary of predictive performance statistics for time to subjective cognitive decline (*SCD*). The table presents cross-validated and hold-out C-indices for single-cohort analyses and the number of features used in each model. Models with the highest average cross-validated C-indices in each cohort analysis are highlighted in bold. The model with the highest hold-out C-index is indicated in *italics*. The "number of features" column includes the number of candidate features selected during cross-validation (shown in brackets) and the subset of features demonstrating significant predictive impact identified through permutation importance analysis (preceding the brackets).

| **Single-cohort analyses** | | | | | | | | | |
|---|---|---|---|---|---|---|---|---|---|
| **Algorithm** | **LuxPARK** | | | **PPMI** | | | **ICEBERG** | | |
| | **Mean (95% CIs)** | **Hold-out C-index** | **Number of features** | **Mean (95% CIs)** | **Hold-out C-index** | **Number of features** | **Mean (95% CIs)** | **Hold-out C-index** | **Number of features** |
| CW-GBoost | ***0.647 (0.629, 0.665)*** | ***0.707*** | ***14 (14)*** | 0.725 (0.678, 0.768) | 0.743 | 13 (26) | 0.665 (0.574, 0.754) | 0.599 | 7 (10) |
| Extra Survival | 0.648 (0.562, 0.734) | 0.685 | 158 (162) | 0.725 (0.707, 0.747) | 0.730 | 14 (14) | 0.773 (0.711, 0.832) | 0.595 | 91 (92) |
| Survival GBoost | 0.664 (0.638, 0.687) | 0.679 | 26 (51) | **0.756 (0.728, 0.782)** | **0.700** | **22 (50)** | 0.714 (0.620, 0.797) | 0.392 | 21 (28) |
| LSVM | 0.654 (0.594, 0.703) | 0.624 | 11 (11) | 0.737 (0.722, 0.753) | 0.722 | 26 (26) | 0.719 (0.562, 0.876) | 0.603 | 5 (5) |
| NLSVM | 0.640 (0.591, 0.688) | 0.640 | 11 (11) | ***0.742 (0.727, 0.761)*** | ***0.762*** | ***31 (31)*** | 0.664 (0.498, 0.845) | 0.477 | 10 (10) |
| Penalized Cox | 0.615 (0.521, 0.692) | 0.615 | 1 (3) | 0.752 (0.727, 0.775) | 0.724 | 15 (48) | 0.623 (0.431, 0.802) | 0.579 | 2 (2) |
| Survival RF | **0.689 (0.630, 0.747)** | **0.660** | **99 (123)** | 0.715 (0.684, 0.748) | 0.739 | 11 (12) | **0.811 (0.738, 0.904)** | **0.549** | **42 (61)** |
| Survival Trees | 0.661 (0.610, 0.710) | 0.608 | 17 (28) | 0.693 (0.678, 0.707) | 0.582 | 7 (17) | ***0.723 (0.541, 0.905)*** | ***0.604*** | ***2 (2)*** |

**Supplementary Table 5. Predictive performance metrics for *PD-MCI* classification in multi-cohort analyses.** Summary of predictive performance metrics for mild cognitive impairment (*PD-MCI*) classification in the multi-cohort analyses, including the cross-cohort analysis using training and validation data from all cohorts, and the leave-one-cohort-out analyses with ICEBERG, PPMI and LuxPARK, respectively, used as the hold-out test set cohorts. The table presents cross-validated and hold-out AUC values and the number of features used in each model. Models with the highest average cross-validated AUC scores in each cohort analysis are highlighted in bold. The model with the highest hold-out AUC score is indicated in *italics*. The “number of features” column includes the number of candidate features selected during cross-validation (shown in brackets) and the subset of features demonstrating significant predictive impact identified through permutation importance analysis (preceding the brackets).

| **Multi-cohort analyses** | | | | | | | | | | | | |
|---|---|---|---|---|---|---|---|---|---|---|---|---|
| | **Cross-Cohort** | | | **Leave-ICEBERG-out** | | | **Leave-PPMI-out** | | | **Leave-LuxPARK-out** | | |
| **Algorithm** | **Mean (95% CIs)** | **Hold -out AUC** | **Number of features** | **Mean (95% CIs)** | **Hold -out AUC** | **Number of features** | **Mean (95% CIs)** | **Hold -out AUC** | **Number of features** | **Mean (95% CIs)** | **Hold -out AUC** | **Number of features** |
| AdaBoost | 0.678 (0.642, 0.729) | 0.670 | 2 (2) | 0.673 (0.635, 0.712) | 0.530 | 3 (5) | **0.699 (0.643, 0.740)** | **0.629** | **2 (7)** | 0.680 (0.660, 0.702) | 0.651 | 2 (7) |
| CART | 0.687 (0.647, 0.736) | 0.632 | 2 (2) | 0.675 (0.634, 0.703) | 0.595 | 4 (8) | 0.668 (0.617, 0.720) | 0.523 | 1 (1) | 0.677 (0.644, 0.708) | 0.653 | 2 (3) |
| CatBoost | **0.691 (0.673, 0.716)** | **0.648** | **5 (7)** | 0.652 (0.635, 0.670) | 0.544 | 8 (9) | 0.683 (0.641, 0.735) | 0.602 | 5 (13) | ***0.685 (0.647, 0.722)*** | ***0.654*** | ***12 (26)*** |
| C4.5 | 0.620 (0.578, 0.665) | 0.551 | 4 (7) | 0.661 (0.633, 0.683) | 0.489 | 3 (5) | 0.638 (0.603, 0.667) | 0.516 | 3 (6) | 0.680 (0.663, 0.703) | 0.460 | 2 (3) |
| FIGS | 0.676 (0.648, 0.718) | 0.632 | 2 (2) | **0.679 (0.631, 0.714)** | **0.446** | **1 (5)** | 0.681 (0.641, 0.730) | 0.523 | 1 (1) | **0.689 (0.664, 0.716)** | **0.651** | **2 (2)** |
| GOSDT-GUESSES | 0.663 (0.610, 0.723) | 0.627 | 22 (34) | 0.606 (0.551, 0.651) | 0.533 | 39 (65) | 0.658 (0.635, 0.685) | 0.489 | 17 (34) | 0.621 (0.584, 0.652) | 0.588 | 16 (18) |
| GBoost | 0.688 (0.645, 0.746) | 0.621 | 14 (32) | ***0.666 (0.638, 0.692)*** | ***0.604*** | ***18 (35)*** | 0.694 (0.656, 0.732) | 0.513 | 4 (9) | 0.673 (0.637, 0.710) | 0.630 | 12 (39) |
| HS | 0.676 (0.648, 0.718) | 0.632 | 2 (2) | **0.679 (0.631, 0.714)** | **0.446** | **1 (5)** | 0.681 (0.641, 0.730) | 0.523 | 1 (1) | **0.689 (0.664, 0.716)** | **0.651** | **2 (2)** |
| XGBoost | ***0.672 (0.621, 0.729)*** | ***0.671*** | ***48 (69)*** | 0.646 (0.631, 0.660) | 0.561 | 36 (63) | 0.694 (0.659, 0.733) | 0.553 | 39 (48) | 0.670 (0.641, 0.704) | 0.650 | 17 (25) |

**Supplementary Table 6. Predictive performance metrics for time-to-*PD-MCI* in multi-cohort analyses.** Summary of predictive performance metrics for time to mild cognitive impairment (*PD-MCI*) for the multi-cohort analyses, including the cross-cohort analysis using training and validation data from all cohorts, and the leave-one-cohort-out analyses with ICEBERG, PPMI and LuxPARK, respectively, used as the hold-out test set cohorts. The table presents cross-validated and hold-out C-indices and the number of features used in each model. Models with the highest average cross-validated C-indices in each cohort analysis are highlighted in bold. The model with the highest hold-out C-index is indicated in *italics*. The "number of features" column includes the number of candidate features selected during cross-validation (shown in brackets) and the subset of features demonstrating significant predictive impact identified through permutation importance analysis (preceding the brackets).

**Multi-cohort analyses**

| Algorithm | Cross-Cohort | | | Leave-ICEBERG-out | | | Leave-PPMI-out | | | Leave-LuxPARK-out | | |
|---|---|---|---|---|---|---|---|---|---|---|---|---|
| | **Mean (95% CIs)** | **Hold-out C-index** | **Number of features** | **Mean (95% CIs)** | **Hold-out C-index** | **Number of features** | **Mean (95% CIs)** | **Hold-out C-index** | **Number of features** | **Mean (95% CIs)** | **Hold-out C-index** | **Number of features** |
| CW-GBoost | 0.598 (0.572, 0.627) | 0.637 | 4 (5) | ***0.646 (0.569, 0.694)*** | ***0.633*** | ***1 (2)*** | ***0.638 (0.611, 0.666)*** | ***0.643*** | ***18 (27)*** | 0.691 (0.684, 0.704) | 0.643 | 12 (23) |
| Extra Survival | 0.583 (0.561, 0.605) | 0.592 | 12 (12) | 0.598 (0.580, 0.618) | 0.483 | 13 (13) | 0.658 (0.611, 0.707) | 0.601 | 137 (138) | 0.680 (0.673, 0.686) | 0.627 | 20 (20) |
| Survival GBoost | ***0.577 (0.520, 0.633)*** | ***0.651*** | ***11 (24)*** | 0.621 (0.589, 0.650) | 0.563 | 9 (18) | **0.670 (0.637, 0.709)** | **0.630** | **27 (61)** | **0.694 (0.682, 0.711)** | **0.645** | **25 (48)** |
| LSVM | 0.590 (0.556, 0.617) | 0.570 | 29 (29) | 0.610 (0.565, 0.655) | 0.500 | 68 (68) | 0.650 (0.621, 0.673) | 0.559 | 33 (33) | 0.687 (0.675, 0.701) | 0.648 | 59 (59) |
| NLSVM | 0.589 (0.547, 0.642) | 0.615 | 40 (40) | 0.628 (0.599, 0.654) | 0.506 | 53 (53) | 0.628 (0.594, 0.667) | 0.560 | 53 (53) | ***0.693 (0.676, 0.708)*** | ***0.661*** | ***58 (58)*** |
| Penalized Cox | **0.610 (0.585, 0.636)** | **0.627** | **2 (3)** | **0.647 (0.628, 0.670)** | **0.512** | **1 (1)** | 0.617 (0.585, 0.652) | 0.610 | 1 (2) | ***0.663 (0.648, 0.677)*** | ***0.661*** | ***2 (2)*** |
| Survival RF | 0.562 (0.533, 0.591) | 0.630 | 6 (6) | 0.620 (0.596, 0.636) | 0.483 | 11 (12) | 0.665 (0.629, 0.703) | 0.641 | 8 (8) | 0.681 (0.674, 0.687) | 0.658 | 22 (22) |
| Survival Trees | 0.591 (0.569, 0.613) | 0.615 | 14 (28) | 0.617 (0.601, 0.636) | 0.525 | 1 (1) | 0.641 (0.612, 0.680) | 0.573 | 5 (16) | 0.648 (0.638, 0.665) | 0.620 | 8 (15) |

**Supplementary Table 7. Predictive performance metrics for *SCD* classification in multi-cohort analyses.** Summary of predictive performance statistics for subjective cognitive decline (*SCD*) classification, including the cross-cohort analysis using training and validation data from all cohorts, and the leave-one-cohort-out analyses with ICEBERG, PPMI and LuxPARK, respectively, used as the hold-out test set cohorts. The table presents cross-validated and hold-out AUC values for multi-cohort analyses and the number of features used in each model. Models with the highest average cross-validated AUC scores in each cohort analysis are highlighted in bold. The model with the highest hold-out AUC score is indicated in *italics*.. The “number of features” column includes the number of candidate features selected during cross-validation (shown in brackets) and the subset of features demonstrating significant predictive impact identified through permutation importance analysis (preceding the brackets).

| **Multi-cohort analyses** | | | | | | | | | | | | |
|---|---|---|---|---|---|---|---|---|---|---|---|---|
| **Algorithm** | **Cross-Cohort** | | | **Leave-ICEBERG-out** | | | **Leave-PPMI-out** | | | **Leave-LuxPARK-out** | | |
| | **Mean (95% CIs)** | **Hold -out AUC** | **Number of features** | **Mean (95% CIs)** | **Hold -out AUC** | **Number of features** | **Mean (95% CIs)** | **Hold -out AUC** | **Number of features** | **Mean (95% CIs)** | **Hold -out AUC** | **Number of features** |
| AdaBoost | 0.699 (0.667, 0.732) | 0.705 | 8 (16) | 0.710 (0.690, 0.729) | 0.545 | 6 (13) | **0.662 (0.608, 0.712)** | **0.590** | **8 (15)** | ***0.676 (0.644, 0.706)*** | ***0.625*** | ***3 (7)*** |
| CART | 0.683 (0.651, 0.730) | 0.663 | 7 (15) | 0.704 (0.685, 0.724) | 0.536 | 1 (2) | 0.646 (0.599, 0.685) | 0.665 | 4 (6) | 0.667 (0.637, 0.697) | 0.597 | 29 (50) |
| CatBoost | ***0.702 (0.659, 0.740)*** | ***0.719*** | ***14 (23)*** | 0.716 (0.689, 0.747) | 0.527 | 7 (8) | 0.654 (0.620, 0.687) | 0.610 | 7 (12) | 0.687 (0.655, 0.710) | 0.577 | 6 (15) |
| C4.5 | 0.657 (0.629, 0.686) | 0.671 | 5 (13) | 0.689 (0.649, 0.726) | 0.536 | 3 (5) | 0.604 (0.570, 0.645) | 0.559 | 8 (13) | 0.685 (0.649, 0.713) | 0.560 | 2 (3) |
| FIGS | 0.682 (0.650, 0.714) | 0.686 | 3 (5) | 0.710 (0.682, 0.736) | 0.482 | 4 (6) | ***0.614 (0.578, 0.646)*** | ***0.705*** | ***4 (7)*** | 0.677 (0.651, 0.695) | 0.558 | 1 (1) |
| GOSDT-GUESSES | 0.652 (0.629, 0.675) | 0.639 | 30 (55) | ***0.654 (0.631, 0.687)*** | ***0.607*** | ***16 (16)*** | 0.656 (0.618, 0.695) | 0.612 | 28 (42) | 0.631 (0.588, 0.665) | 0.575 | 40 (64) |
| GBoost | **0.703 (0.665, 0.742)** | **0.717** | **10 (14)** | **0.729 (0.690, 0.765)** | **0.545** | **15 (29)** | 0.643 (0.623, 0.669) | 0.583 | 12 (23) | **0.691 (0.661, 0.726)** | **0.575** | **12 (28)** |
| HS | 0.682 (0.650, 0.714) | 0.686 | 3 (5) | 0.710 (0.682, 0.736) | 0.482 | 4 (6) | ***0.614 (0.578, 0.646)*** | ***0.705*** | ***4 (7)*** | 0.677 (0.651, 0.695) | 0.558 | 1 (1) |
| XGBoost | 0.678 (0.647, 0.715) | 0.697 | 35 (48) | 0.702 (0.681, 0.729) | 0.509 | 48 (54) | 0.636 (0.591, 0.674) | 0.697 | 31 (65) | 0.676 (0.659, 0.695) | 0.571 | 46 (50) |

**Supplementary Table 8. Predictive performance metrics for time-to-*SCD* in multi-cohort analyses.** Summary of predictive performance statistics for time to subjective cognitive decline (*SCD*) for the multi-cohort analyses, including the cross-cohort analysis using training and validation data from all cohorts, and the leave-one-cohort-out analyses with ICEBERG, PPMI and LuxPARK, respectively, used as the hold-out test set cohorts. The table presents cross-validated and hold-out C-indices for multi-cohort analyses and the number of features used in each model. Models with the highest average cross-validated C-indices in each cohort analysis are highlighted in bold. The model with the highest hold-out C-index is indicated in *italics*. The "number of features" column includes the number of candidate features selected during cross-validation (shown in brackets) and the subset of features demonstrating significant predictive impact identified through permutation importance analysis (preceding the brackets).

**Multi-cohort analyses**

| Algorithm | Cross-Cohort | | | Leave-ICEBERG-out | | | Leave-PPMI-out | | | Leave-LuxPARK-out | | |
|---|---|---|---|---|---|---|---|---|---|---|---|---|
| | Mean (95% CIs) | Hold-out C-index | Number of features | Mean (95% CIs) | Hold-out C-index | Number of features | Mean (95% CIs) | Hold-out C-index | Number of features | Mean (95% CIs) | Hold-out C-index | Number of features |
| CW-GBoost | **0.729 (0.711, 0.754)** | **0.683** | **14 (19)** | 0.736 (0.716, 0.755) | 0.605 | 11 (18) | **0.671 (0.650, 0.697)** | **0.680** | **5 (9)** | ***0.755 (0.736, 0.774)*** | ***0.644*** | ***12 (23)*** |
| Extra Survival | 0.726 (0.713, 0.739) | 0.700 | 164 (165) | 0.734 (0.714, 0.754) | 0.588 | 164 (165) | 0.640 (0.608, 0.684) | 0.675 | 18 (18) | 0.753 (0.723, 0.788) | 0.616 | 129 (129) |
| Survival GBoost | 0.722 (0.697, 0.746) | 0.704 | 12 (30) | 0.744 (0.718, 0.773) | 0.585 | 14 (25) | 0.669 (0.648, 0.700) | 0.676 | 10 (21) | 0.760 (0.732, 0.800) | 0.633 | 24 (45) |
| LSVM | 0.714 (0.696, 0.742) | 0.691 | 180 (180) | 0.738 (0.725, 0.755) | 0.625 | 53 (53) | 0.658 (0.632, 0.679) | 0.617 | 151 (151) | 0.758 (0.733, 0.785) | 0.613 | 49 (49) |
| NLSVM | ***0.729 (0.700, 0.760)*** | ***0.718*** | ***48 (48)*** | 0.726 (0.704, 0.748) | 0.597 | 49 (49) | 0.651 (0.626, 0.669) | 0.658 | 151 (151) | 0.762 (0.735, 0.793) | 0.607 | 56 (56) |
| Penalized Cox | 0.708 (0.692, 0.722) | 0.660 | 13 (29) | **0.748 (0.739, 0.757)** | **0.641** | **17 (28)** | 0.657 (0.632, 0.694) | 0.550 | 1 (32) | **0.774 (0.744, 0.806)** | **0.637** | **83 (114)** |
| Survival RF | 0.727 (0.714, 0.741) | 0.697 | 139 (149) | 0.729 (0.704, 0.755) | 0.603 | 13 (13) | *0.659 (0.639, 0.686)* | *0.698* | *68 (106)* | 0.750 (0.715, 0.795) | 0.626 | 115 (120) |
| Survival Trees | 0.674 (0.662, 0.691) | 0.657 | 7 (9) | 0.692 (0.664, 0.717) | 0.659 | 8 (10) | 0.640 (0.615, 0.667) | 0.510 | 19 (35) | 0.691 (0.663, 0.722) | 0.602 | 3 (6) |

**Supplementary Figure 1. Comparison of cross-validated AUC scores for *PD-MCI* classification models.** A comparison of cross-validated AUC scores and probabilities of superior predictive performance for the mild cognitive impairment (*PD-MCI*) classification model with the highest average cross-validated AUCs in the single- and multi-cohort analyses. The upper section presents boxplots of cross-validated AUC scores for each cohort, with the dotted line marking hold-out AUC scores. The lower section shows probabilities of one cohort outperforming another, with arrows indicating the direction of higher predictive performance.

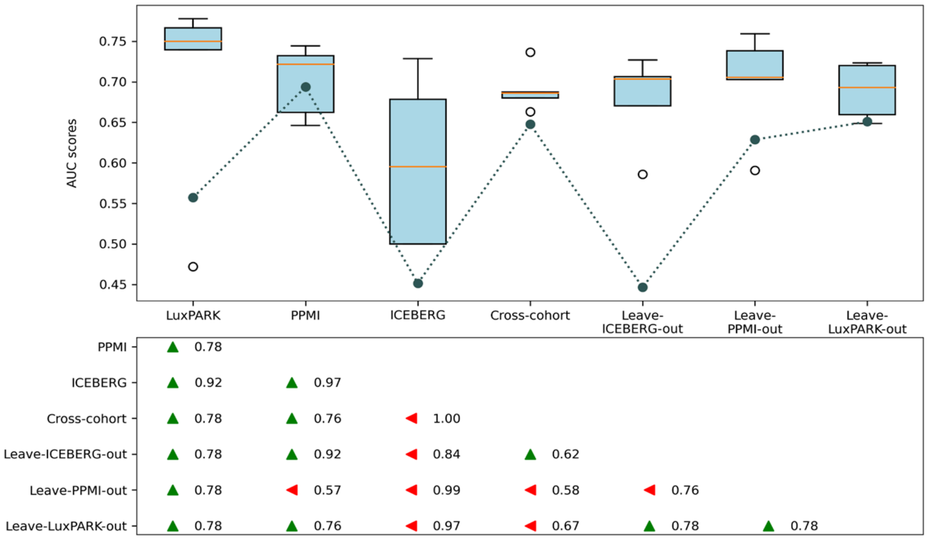


**Supplementary Figure 2. Comparison of cross-validated C-indices for time-to-*PD-MCI* models.** A comparison of cross-validated C-indices and probabilities of superior predictive performance for the time-to-*PD-MCI* (mild cognitive impairment) model with the highest average cross-validated C-indices in the single- and multi-cohort analyses. The upper section presents boxplots of cross-validated C-indices for each cohort, with the dotted line marking hold-out C-indices. The lower section shows probabilities of one cohort outperforming another, with arrows indicating the direction of higher predictive performance.

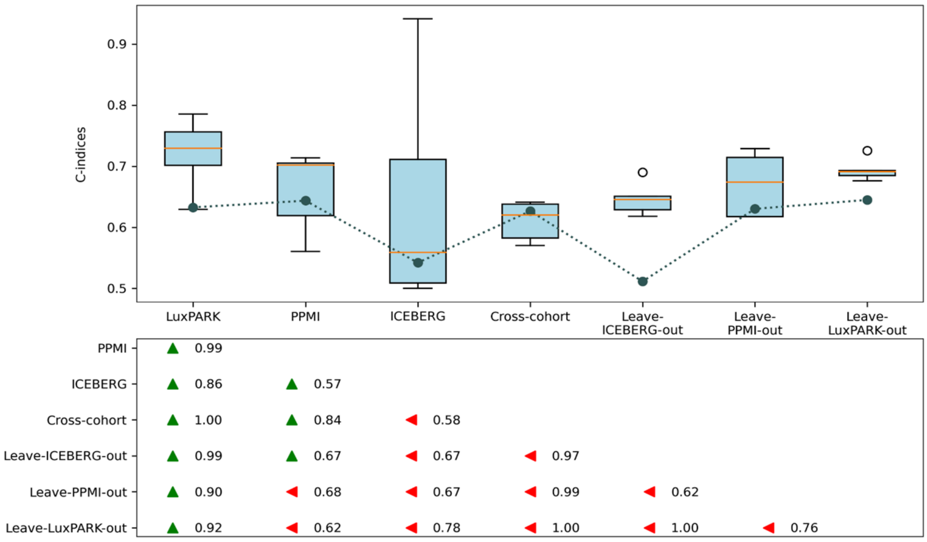

**Supplementary Figure 3. Comparison of cross-validated AUC scores for *SCD* classification.** A comparison of cross-validated AUC scores and probabilities for superior predictive performance for the subjective cognitive decline (*SCD*) classification model with the highest average cross-validated AUCs in the single- and multi-cohort analyses. The upper section presents boxplots of cross-validated AUC scores for each cohort, with the dotted line marking hold-out AUC scores. The lower section shows probabilities of one cohort outperforming another, with arrows indicating the direction of higher predictive performance.

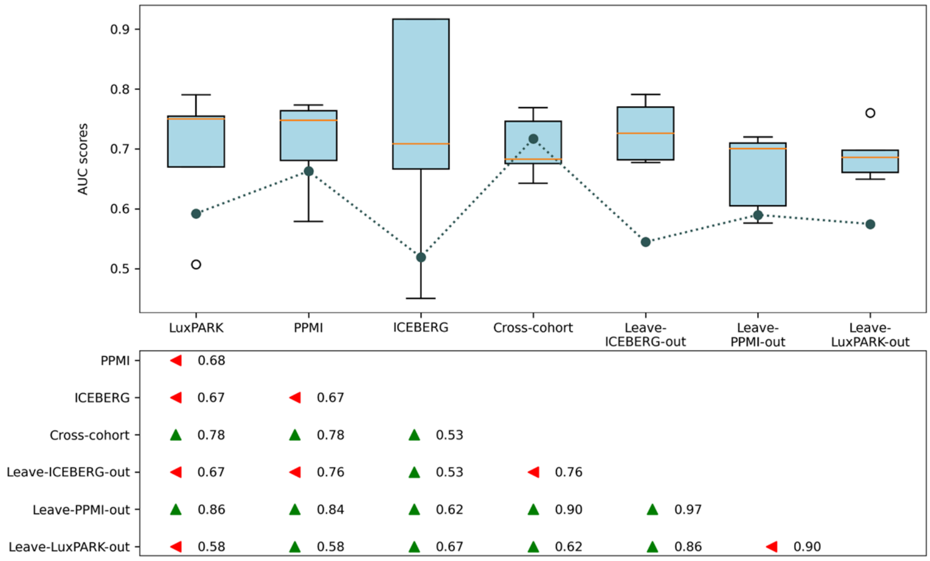


**Supplementary Figure 4. Comparison of cross-validated C-indices for time-to-*SCD* models.** A comparison of cross-validated C-indices and probabilities for superior predictive performance for the time-to-*SCD* (subjective cognitive decline) model with the highest average cross-validated C-indices in the single- and multi-cohort analyses. The upper section presents boxplots of cross-validated C-indices for each cohort, with the dotted line marking hold-out C-indices. The lower section shows probabilities of one cohort outperforming another, with arrows indicating the direction of higher predictive performance.

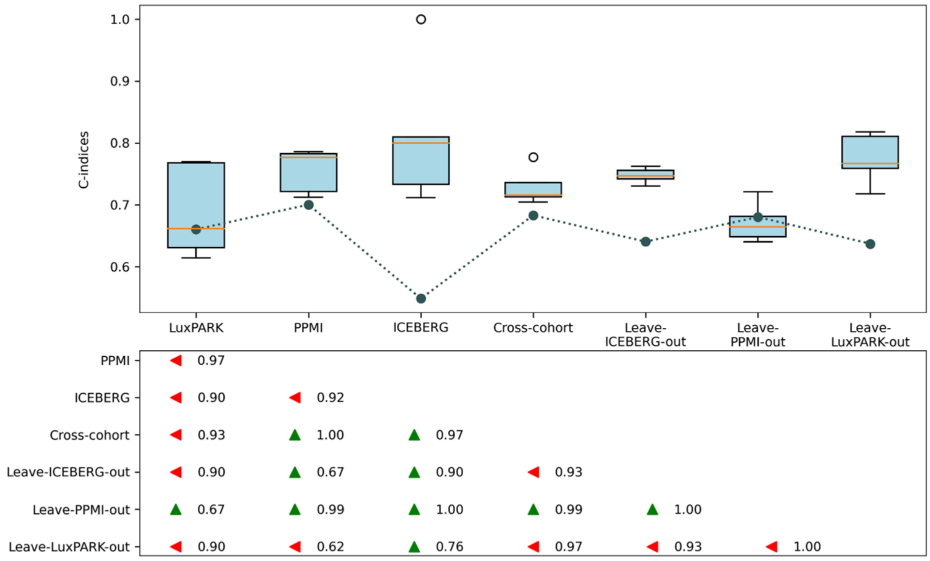

## Stability of the predictive models for predicting the occurrence of cognitive impairment

**Supplementary Figure 5**. **Stability analysis of *PD-MCI* classification models**. Stability analysis for the models for predicting mild cognitive impairment (*PD-MCI*) in Parkinson's disease (PD) across different algorithms and cohort studies. The stability of the model is evaluated by calculating the standard deviations of the area under the curve (AUC) values across the cross-validation cycles. A lower standard deviation (SD) indicates a higher stability of the predictive models.

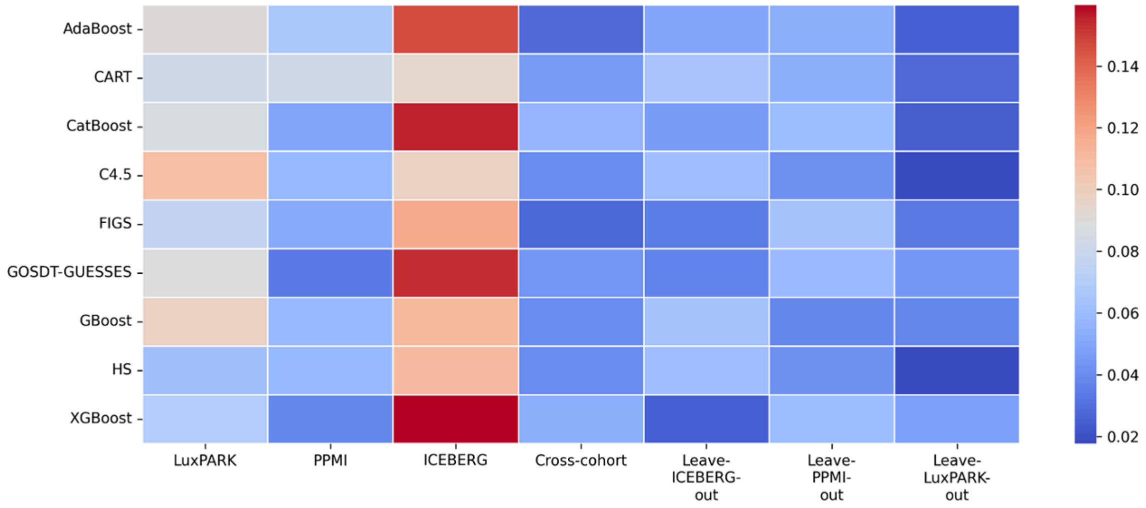


**Supplementary Figure 6. Stability analysis of time-to-*PD-MCI* models.** Stability analysis of time to mild cognitive impairment (*PD-MCI*) models in Parkinson's disease (PD) across different algorithms and cohort studies. The stability of the model is evaluated by calculating the standard deviations of the C-indices across the cross-validation cycles. A lower standard deviation (SD) indicates a higher stability of the predictive models.

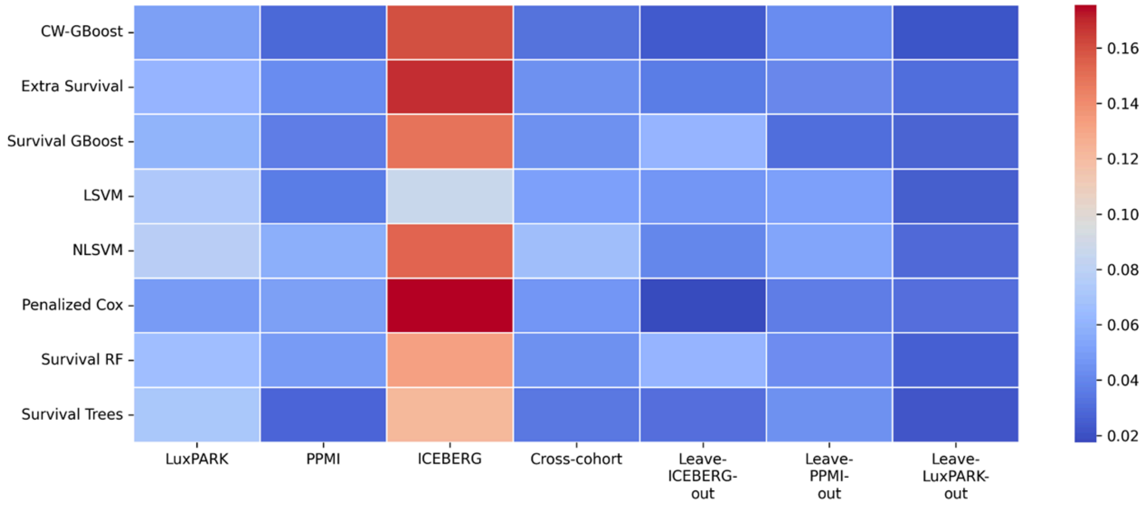

**Supplementary Figure 7. Stability analysis of *SCD* classification models.** Stability analysis of models for predicting subjective cognitive decline (*SCD*) in Parkinson's disease (PD) across different algorithms and cohort studies. The stability of the model is evaluated by calculating the standard deviations of the area under the curve (AUC) values across the cross-validation cycles. A lower standard deviation (SD) indicates a higher stability of the predictive models.

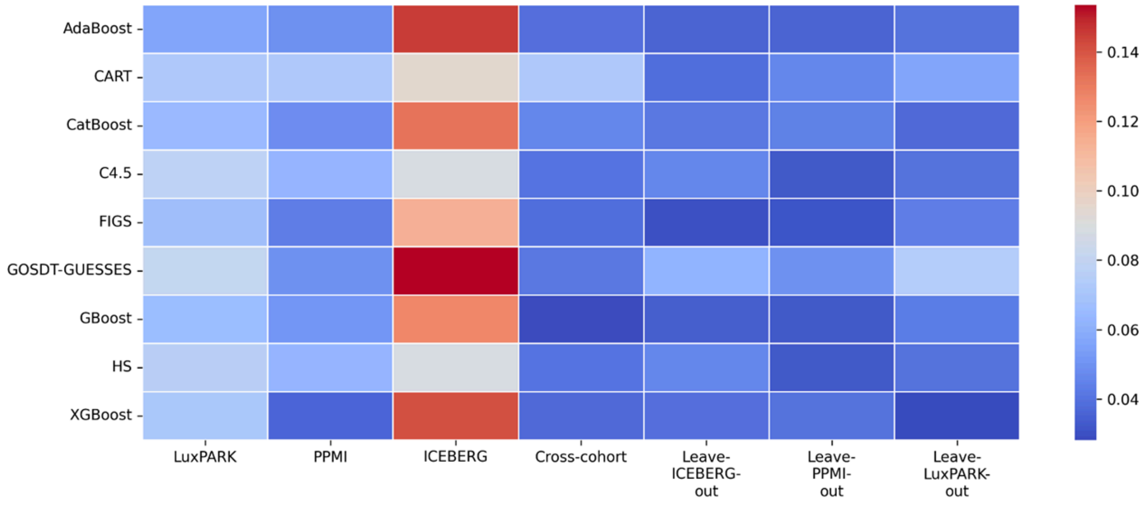


**Supplementary Figure 8. Stability analysis of time-to-*SCD* models.** Stability analysis of time to subjective cognitive decline (*SCD*) models in Parkinson's disease (PD) across different algorithms and cohort studies. The stability of the model is evaluated by calculating the standard deviations of the C-indices across the different cross-validation cycles. A lower standard deviation (SD) indicates a higher stability of the predictive models.

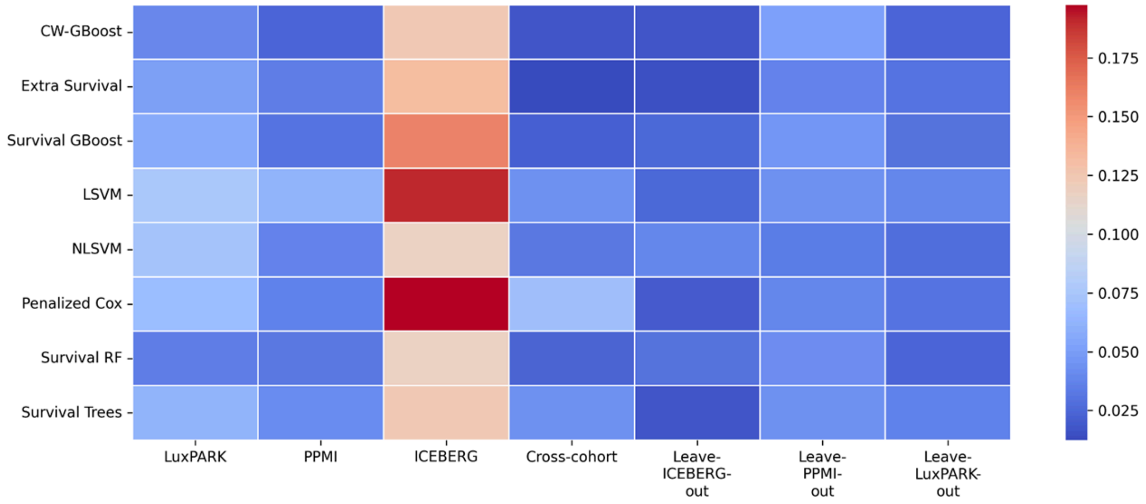

# Model performance statistics for cognitive impairment prognosis: Unnormalized vs. normalized models compared across multiple cohorts

**Supplementary Table 9. Significance testing of hold-out predictive metrics between normalized and unnormalized models.** Comparison of the statistical significance in hold-out predictive metrics (AUC/C-index) between the cross-study normalized and unnormalized models with the highest average cross-validated AUC/C-index. The type of normalization applied is indicated in the "Normalization" column. The statistical significance of the differences was assessed using DeLong's test for classification (top) and a one-shot nonparametric test for time-to-event analysis (bottom).

| | Mild cognitive impairment (*PD-MCI*) | | Subjective cognitive decline (*SCD*) | |
|---|---|---|---|---|
| **Cohort** | **Normalized vs Unnormalized** | **Normalization** | **Normalized vs Unnormalized** | **Normalization** |
| **Classification:** | | | | |
| Cross-cohort | 0.400 | Ratio-A | 0.626 | Standardize |
| Leave-ICEBERG-out | 0.459 | Ratio-A | 0.683 | Mean-centering |
| Leave-PPMI-out | 0.021 | Mean-centering | 0.028 | ComBat |
| Leave-LuxPARK-out | 0.170 | M-ComBat | 0.892 | ComBat |
| **Time-to-event:** | | | | |
| Cross-cohort | 0.051 | M-ComBat | 0.207 | Mean-centering |
| Leave-ICEBERG-out | 1.000 | ComBat | 0.037 | Ratio-A |
| Leave-PPMI-out | 0.121 | ComBat | 3.8E-05 | Mean-centering |
| Leave-LuxPARK-out | 0.065 | ComBat | 0.055 | Standardize |

**Supplementary Table 10. Predictive performance metrics between normalized and unnormalized models.** Evaluation of the predictive performance for mild cognitive impairment (*PD-MCI*) and subjective cognitive decline (*SCD*) prognostic models, including *PD-MCI*/*SCD* classification and time-to-*PD-MCI*/*SCD* analysis models. This evaluation provides cross-validated and hold-out AUC values and C-indices for normalized and unnormalized models. The "number of features" column indicates both the total number of candidate features selected during cross-validation (in brackets) and the number of features with significant predictive impact, as determined by permutation importance analysis (preceding the brackets).

| | **Cross-Cohort** | | | **Leave-ICEBERG-out** | | | **Leave-PPMI-out** | | | **Leave-LuxPARK-out** | | |
|---|---|---|---|---|---|---|---|---|---|---|---|---|
| | **Mean (95% CIs)** | **Hold-out AUC** | **Number of features** | **Mean (95% CIs)** | **Hold-out AUC** | **Number of features** | **Mean (95% CIs)** | **Hold-out AUC** | **Number of features** | **Mean (95% CIs)** | **Hold-out AUC** | **Number of features** |
| ***PD-MCI* classification:** | | | | | | | | | | | | |
| Normalized | 0.691 (0.673, 0.716) | 0.648 | 5 (7) | 0.679 (0.631, 0.714) | 0.446 | 1 (5) | 0.699 (0.643, 0.740) | 0.629 | 2 (7) | 0.689 (0.664, 0.716) | 0.651 | 2 (2) |
| Un-normalized | 0.669 (0.607, 0.712) | 0.617 | 7 (16) | 0.666 (0.638, 0.692) | 0.604 | 18 (35) | 0.694 (0.656, 0.732) | 0.513 | 4 (9) | 0.680 (0.649, 0.712) | 0.651 | 8 (16) |
| ***SCD* classification:** | | | | | | | | | | | | |
| Normalized | 0.703 (0.665, 0.742) | 0.717 | 10 (14) | 0.710 (0.690, 0.729) | 0.545 | 6 (13) | 0.656 (0.618, 0.695) | 0.612 | 28 (42) | 0.685 (0.638, 0.717) | 0.616 | 8 (17) |
| Un-normalized | 0.702 (0.659, 0.740) | 0.719 | 14 (23) | 0.729 (0.690, 0.765) | 0.545 | 15 (29) | 0.662 (0.608, 0.712) | 0.590 | 8 (15) | 0.691 (0.661, 0.726) | 0.575 | 12 (28) |
| | **Mean (95% CIs)** | **Hold-out C-index** | **Number of features** | **Mean (95% CIs)** | **Hold-out C-index** | **Number of features** | **Mean (95% CIs)** | **Hold-out C-index** | **Number of features** | **Mean (95% CIs)** | **Hold-out C-index** | **Number of features** |
| **Time-to-*PD-MCI*:** | | | | | | | | | | | | |
| Normalized | 0.610 (0.585, 0.636) | 0.627 | 2 (3) | 0.647 (0.628, 0.670) | 0.512 | 1 (1) | 0.670 (0.637, 0.709) | 0.630 | 27 (61) | 0.694 (0.682, 0.711) | 0.645 | 25 (48) |
| Un-normalized | 0.594 (0.540, 0.660) | 0.662 | 1 (2) | 0.631 (0.619, 0.644) | 0.512 | 1 (2) | 0.658 (0.611, 0.707) | 0.601 | 137 (138) | 0.694 (0.681, 0.709) | 0.602 | 72 (110) |
| **Time-to-*SCD*:** | | | | | | | | | | | | |
| Normalized | 0.729 (0.711, 0.754) | 0.683 | 14 (19) | 0.744 (0.718, 0.773) | 0.585 | 14 (25) | 0.671 (0.650, 0.697) | 0.680 | 5 (9) | 0.774 (0.744, 0.806) | 0.637 | 83 (114) |
| Un-normalized | 0.726 (0.713, 0.739) | 0.700 | 164 (165) | 0.748 (0.739, 0.757) | 0.641 | 17 (28) | 0.658 (0.641, 0.685) | 0.605 | 11 (21) | 0.762 (0.728, 0.799) | 0.655 | 19 (33) |

# Comparative baseline clinical feature analysis across cohorts

**Supplementary Table 11. Comparative analysis of baseline features mean differences across cohorts.** Comparative analysis of baseline features' mean differences between the LuxPARK, PPMI, and ICEBERG cohorts. The *p*-values highlight statistically significant differences in predictor averages between specific cohort pairs, revealing variations in predictor distributions specific to each cohort in mild cognitive impairment (*PD-MCI*) and subjective cognitive decline (*SCD*) analysis.

| **Predictors** | **LuxPARK vs. PPMI (*p*-values)** | **LuxPARK vs. ICEBERG (*p*-values)** | **PPMI vs. ICEBERG (*p*-values)** | ***p*-values** |
|---|---|---|---|---|
| **Mild cognitive impairment (*PD-MCI*)** | | | | |
| Age at PD diagnosis | 1.72 (0.008) | 0.71 (1.00) | -1.01 (0.858) | 1.10E-02 |
| Disease duration since PD diagnosis (years) | 3.29 (4.72E-32) | 3.73 (1.55E-16) | 0.44 (0.383) | 1.40E-36 |
| Weight (kg) | 0.45 (1.00) | 5.80 (0.002) | 5.35 (0.005) | 2.70E-03 |
| Height (cm) | -2.9 (1.14E-05) | -2.04 (0.165) | 0.86 (1.00) | 2.05E-05 |
| BMI (kg/m$^2$) | 1.04 (2.20E-04) | 2.67 (3.40E-08) | 1.63 (0.002) | 6.43E-09 |
| MDS-UPDRS Part I score | 0.82 (2.36E-15) | 0.23 (0.108) | -0.59 (5.82E-05) | 6.22E-15 |
| MDS-UPDRS Part II score | 8.18 (9.41E-107) | 2.01 (1.00) | -6.16 (4.48E-33) | 7.83E-115 |
| MDS-UPDRS Part III (ON) score | 5.77 (1.14E-37) | 4.32 (5.56E-04) | -1.45 (4.91E-04) | 5.82E-37 |
| SCOPA-AUT total score | 1.37 (8.65E-08) | 0.48 (0.853) | -0.89 (0.101) | 1.65E-07 |
| **Subjective cognitive decline (*SCD*)** | | | | |
| Age at PD diagnosis | 1.63 (0.05) | -0.35 (1.00) | -1.99 (0.14) | 2.25E-02 |
| Disease duration since PD diagnosis (years) | 3.43 (5.71E-31) | 3.99 (5.98E-19) | 0.56 (0.26) | 3.14E-36 |
| Weight (kg) | 0.37 (1.00) | 5.49 (0.005) | 5.12 (0.011) | 5.90E-03 |
| Height (cm) | -2.24 (0.003) | -1.75 (0.296) | 0.48 (1.00) | 4.40E-03 |
| BMI (kg/m$^2$) | 0.82 (0.013) | 2.47 (7.55E-07) | 1.65 (0.002) | 9.30E-07 |
| MDS-UPDRS Part I score | 8.68 (2.27E-86) | 2.42 (0.598) | -6.26 (1.16E-29) | 1.41E-93 |
| MDS-UPDRS Part II score | 6.36 (6.04E-37) | 4.88 (1.81E-05) | -1.48 (9.53E-04) | 3.24E-36 |
| MDS-UPDRS Part III (ON) score | 19.49 (1.45E-35) | 9.81 (3.32E-06) | -9.67 (6.73E-08) | 7.00E-36 |
| SCOPA-AUT total score | 0.58 (8.54E-07) | 0.74 (6.16E-06) | 0.16 (0.344) | 1.50E-08 |

## SHAP value for the cross-cohort analysis

**Supplementary Figure 9**: **SHAP value plot revealing key predictors' influence on time-to-*PD-MCI* prediction in the cross-cohort analysis.** The plot illustrates how three key predictors affect the time to development of mild cognitive impairment (*PD-MCI*). Each point represents an individual patient, with colors indicating the predictor's value (red = high, blue = low). Higher SHAP values (right side) indicate faster progression to *PD-MCI*. The Benton Judgment of Line Orientation score shows the strongest association, with higher scores (red) associated with longer time to *PD-MCI*. Age at PD diagnosis demonstrates the most pronounced distribution, with later onset (red) strongly predicting faster progression to *PD-MCI*. The SCOPA-AUT Total score shows only a very modest effect, with higher autonomic dysfunction scores slightly accelerating time to cognitive impairment.

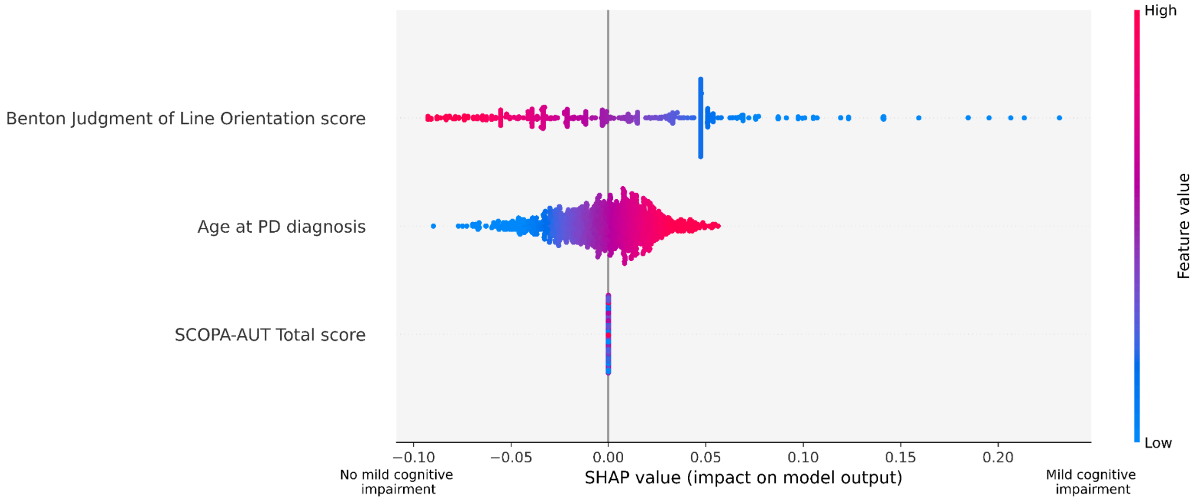


**Supplementary Figure 10. SHAP value plot revealing key predictors' influence on time-to-*SCD* prediction in the cross-cohort analysis.** The plot illustrates how three key predictors affect the time to development of subjective cognitive decline (*SCD*). Each point represents an individual patient, with colors indicating the predictor's value (red = high, blue = low). Higher SHAP values (right side) indicate faster progression to *SCD*. The MDS-UPDRS Part I score shows the strongest effect, with higher scores (red) associated with increased *SCD* risk.

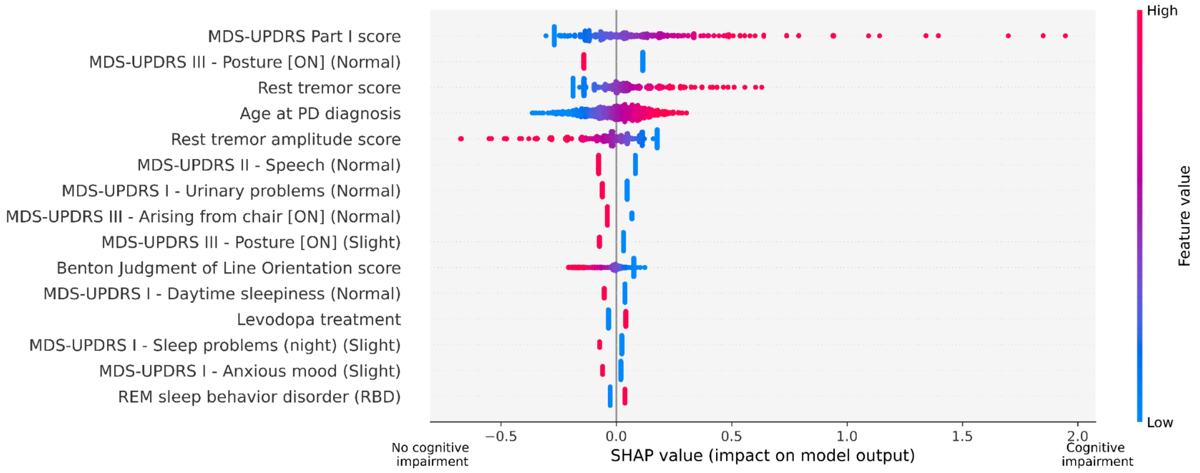

## Correlation analysis of predictors

**Supplementary Table 12. Correlation analysis results for *PD-MCI* predictors in cross-cohort analysis.** The results of the correlation analysis of the *PD-MCI* predictors. The Spearman correlation coefficient was used for continuous or ordinal variable pairs, the point-biserial correlation coefficient was used for continuous or ordinal and binary variable pairs, and the Matthews correlation coefficient (MCC) was used for binary variable pairs. *P*-values in parentheses accompany the correlation coefficients are presented to indicate the statistical significance of the correlation.

| | Age at PD diagnosis | MoCA score | Disease duration | MDS-UPDRS Part I | MDS-UPDRS Part II | MDS-UPDRS Part III (ON) | SCOPA-AUT total score |
|---|---|---|---|---|---|---|---|
| MoCA score | -0.31 (9.1E-17) | | | | | | |
| Benton JLO | -0.13 (8.3E-06) | 0.28 (7.5E-12) | | | | | |
| Disease duration | -0.19 (1.5E-11) | -0.15 (9.8E-05) | -0.16 (5.9E-08) | | | | |
| MDS-UPDRS Part I | 0.03 (3.0E-01) | -0.14 (3.0E-04) | -0.28 (2.6E-21) | 0.32 (7.2E-31) | | | |
| MDS-UPDRS Part II | 0.02 (3.9E-01) | -0.23 (5.4E-10) | -0.21 (2.9E-12) | 0.36 (4.0E-39) | 0.57 (3.2E-108) | | |
| MDS-UPDRS Part III (ON) | 0.17 (2.1E-06) | -0.37 (8.9E-22) | -0.34 (1.2E-18) | 0.23 (2.5E-10) | 0.39 (7.0E-28) | 0.51 (2.9E-49) | |
| SCOPA-AUT total score | 0.15 (9.8E-08) | -0.14 (2.6E-04) | -0.25 (2.8E-17) | 0.36 (3.5E-39) | 0.68 (3.9E-164) | 0.55 (1.3E-96) | 0.39 (1.5E-26) |

**Supplementary Table 13. Correlation between predictors and *PD-MCI/SCD* outcomes in cross-cohort analysis.** The correlation between predictors and the outcome of mild cognitive impairment (*PD-MCI*) and subjective cognitive decline (*SCD*) in the cross-cohort analysis. The correlation is measured using the point-biserial correlation for continuous or ordinal predictors and the Matthews correlation coefficient (MCC) for binary predictors.

| Predictors | Mild cognitive impairment (*PD-MCI*) | | Subjective cognitive decline (*SCD*) | |
|---|---|---|---|---|
| | Correlation | *p*-values | Correlation | *p*-values |
| Age at PD diagnosis | 0.31 | 2.31E-30 | 0.24 | 8.33E-15 |
| Disease duration since PD diagnosis (years) | 0.14 | 1.36E-06 | 0.19 | 2.84E-10 |
| Gender | 0.04 | 2.20E-03 | 0.04 | 3.94E-03 |
| Levodopa treatment | 0.21 | 1.30E-13 | 0.25 | 4.58E-16 |
| Weight (kg) | 0.06 | 4.44E-02 | 0.02 | 5.44E-01 |
| Height (cm) | -0.11 | 1.89E-04 | -0.05 | 9.76E-02 |
| BMI (kg/m$^2$) | 0.14 | 3.51E-07 | 0.05 | 8.49E-02 |
| Hoehn & Yahr stage | 0.20 | 1.70E-12 | 0.29 | 2.65E-22 |
| MOCA score (adjusted for education) | -0.47 | 7.91E-41 | -0.34 | 1.86E-15 |
| Benton Judgment of Line Orientation (JLO) | -0.26 | 4.00E-19 | -0.30 | 3.88E-21 |
| REM sleep behavior disorder (RBD) | 0.11 | 1.74E-04 | 0.20 | 8.05E-10 |
| Initial motor symptom - Resting tremor | -0.06 | 2.43E-02 | -0.13 | 6.26E-05 |
| Initial motor symptom - Rigidity or bradykinesia | -0.09 | 1.21E-03 | -0.10 | 9.71E-04 |
| Axial symptoms | 0.26 | 2.43E-20 | 0.37 | 1.10E-35 |
| Selective axial symptoms | 0.24 | 3.08E-18 | 0.33 | 2.66E-28 |
| Freezing of gait | 0.14 | 3.95E-07 | 0.19 | 4.67E-10 |
| Tremor | 0.08 | 2.23E-02 | 0.08 | 3.91E-02 |
| Rest tremor | 0.09 | 1.30E-02 | 0.04 | 2.70E-01 |
| Rest tremor amplitude | 0.11 | 3.53E-03 | 0.05 | 1.82E-01 |
| Rigidity upper extremities | 0.17 | 2.80E-06 | 0.23 | 3.67E-09 |
| Rigidity lower extremities | 0.16 | 1.07E-05 | 0.19 | 8.32E-07 |
| Total rigidity | 0.20 | 2.57E-08 | 0.25 | 2.30E-10 |
| Bradykinesia | 0.26 | 8.53E-13 | 0.35 | 2.98E-20 |
| Dyskinesia | 0.03 | 3.77E-01 | 0.04 | 2.99E-01 |
| Motor fluctuations | 0.08 | 1.70E-02 | 0.09 | 1.94E-02 |
| MDS-UPDRS Part I score | 0.21 | 1.38E-14 | 0.53 | 6.85E-77 |
| MDS-UPDRS Part II score | 0.21 | 1.46E-14 | 0.40 | 1.46E-41 |
| MDS-UPDRS Part III score (ON) | 0.28 | 4.75E-15 | 0.35 | 8.32E-20 |
| SCOPA-AUT Gastrointestinal (GI) | 0.22 | 1.85E-15 | 0.35 | 9.77E-32 |
| SCOPA-AUT Urinary | 0.12 | 1.90E-05 | 0.29 | 5.41E-22 |
| SCOPA-AUT Cardiovascular | 0.09 | 1.33E-03 | 0.26 | 1.41E-17 |
| SCOPA-AUT Thermoregulatory | 0.02 | 5.22E-01 | 0.18 | 8.58E-09 |
| SCOPA-AUT Sexual dysfunction | 0.04 | 2.61E-01 | 0.11 | 1.29E-02 |
| SCOPA-AUT Total score | 0.21 | 3.98E-14 | 0.43 | 6.27E-47 |
| Family history of PD | -0.08 | 1.09E-02 | -0.13 | 2.56E-05 |
| Pathogenic *LRRK2* variant | -0.14 | 8.55E-06 | -0.22 | 2.53E-11 |
| Pathogenic *GBA* variant | -0.02 | 5.76E-01 | -0.01 | 8.12E-01 |

## Evaluation of predictive models using decision curve and calibration analysis

**Supplementary Figure 11. Bar plot of the area under the positive net benefit curve for the *PD-MCI* classification models in cross-cohort analysis.** Bar plot illustrating the area under the positive net benefit curve for various cross-cohort mild cognitive impairment (*PD-MCI*) classification models. The lines indicate significant differences in the net benefit area across the models. The blue bars show a larger positive net benefit area than negative net benefit, whereas the red bars indicate the opposite. The numbers within the bars represent the difference in net benefit area relative to the “all intervention” strategy, with ↑ indicating a larger area and ↓ a smaller area.

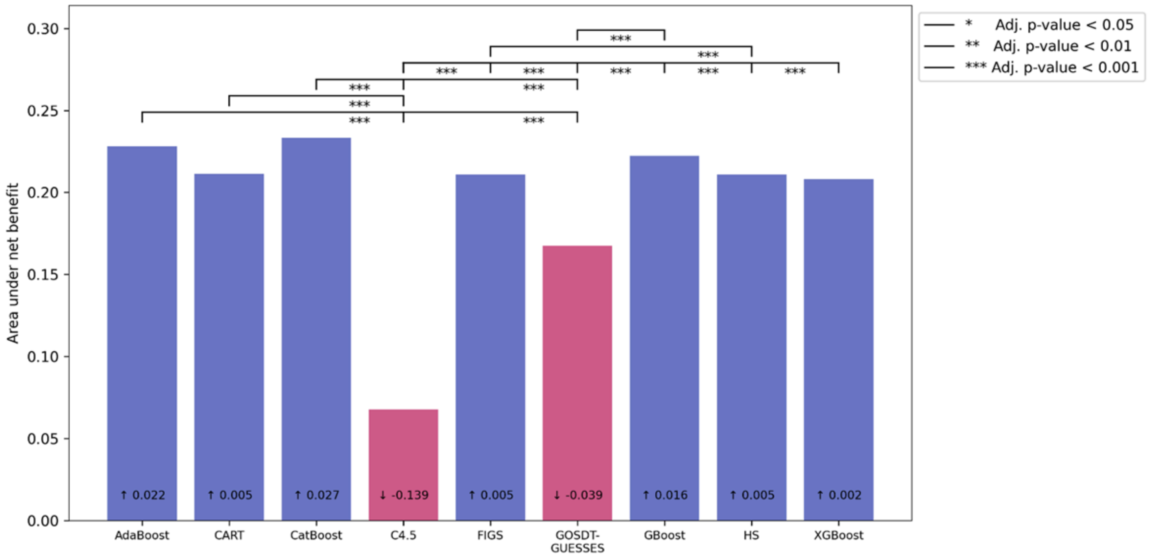


**Supplementary Figure 12. Bar plot of the area under the positive net benefit curve for the time-to-*PD-MCI* models in cross-cohort analysis.** Bar plot illustrating the area under the positive net benefit curve for various cross-cohort time-to-*PD-MCI* (mild cognitive impairment) models. The lines indicate significant differences in the net benefit area across the models. The blue bars show a larger positive net benefit area than the negative net benefit, whereas the red bars indicate the opposite. The numbers within the bars represent the difference in net benefit area relative to the “all intervention” strategy, with ↑ indicating a larger area and ↓ a smaller area.

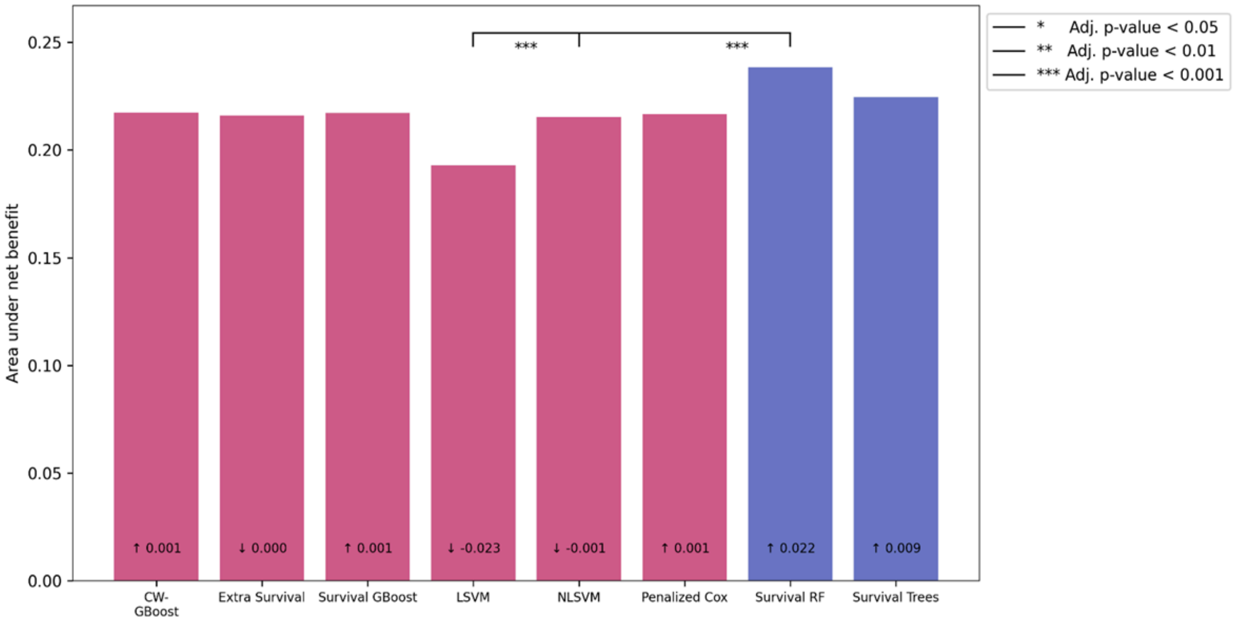

**Supplementary Figure 13. Bar plot of the area under the positive net benefit curve for the *SCD* classification models in cross-cohort analysis.** Bar plot illustrating the area under the positive net benefit curve for various cross-cohort subjective cognitive decline (*SCD*) classification models. The lines indicate significant differences in the net benefit area across the models. The blue bars show a larger positive net benefit area than negative net benefit, whereas the red bars indicate the opposite. The numbers within the bars represent the difference in net benefit area relative to the “all intervention” strategy, with ↑ indicating a larger area and ↓ a smaller area.

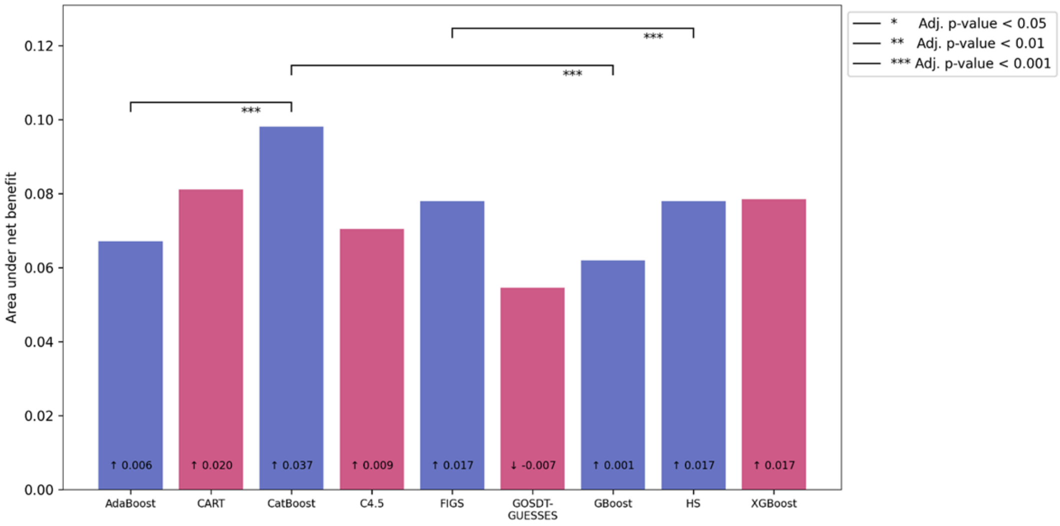


**Supplementary Figure 14. Bar plot of the area under the positive net benefit curve for the time-to-*SCD* models in cross-cohort analysis.** Bar plot illustrating the area under the positive net benefit curve for various cross-cohort time-to-*SCD* (subjective cognitive decline) models. The lines indicate significant differences in the net benefit area across the models. The blue bars show a larger positive net benefit area than the negative net benefit, whereas the red bars indicate the opposite. The numbers within the bars represent the difference in net benefit area relative to the “all intervention” strategy, with ↑ indicating a larger area and ↓ a smaller area.

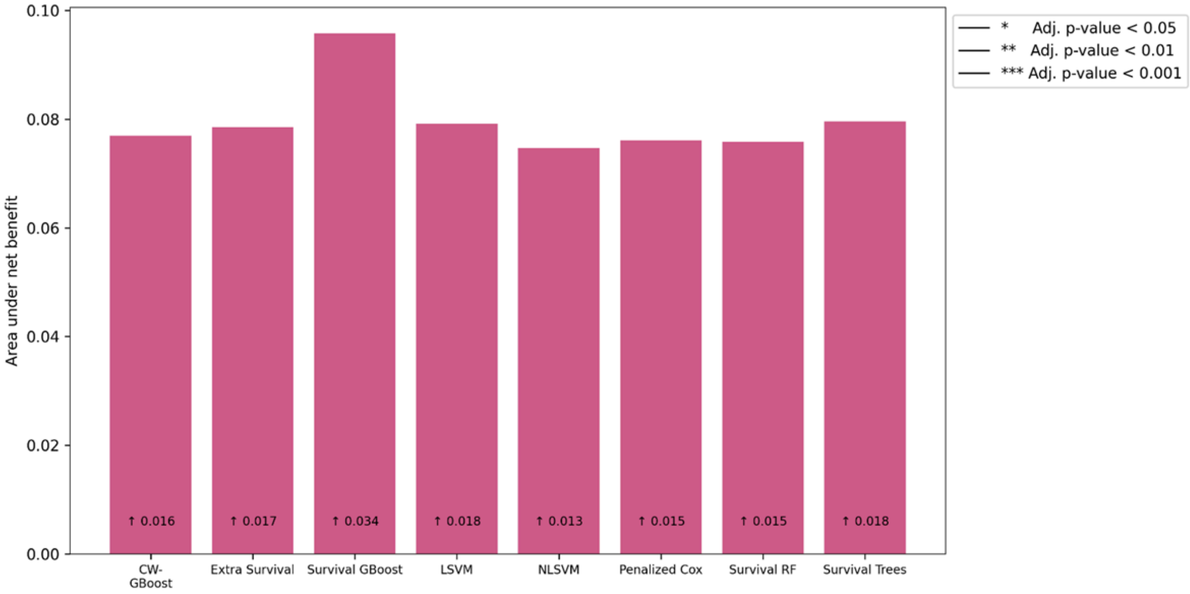

**Supplementary Table 14. Calibration analysis for *PD-MCI* and *SCD* analyses.** The calibration analysis results for mild cognitive impairment (*PD-MCI*) and subjective cognitive decline (*SCD*) models in both classification and time-to-event analyses in cross-cohort analysis. The calibration slope and mean squared error (MSE) illustrate the agreement between predicted probabilities and observed outcomes.

| Classification | | | | | Time-to-event | | | | |
|---|---|---|---|---|---|---|---|---|---|
| **Model** | ***PD-MCI*** | | ***SCD*** | | **Model** | ***PD-MCI*** | | ***SCD*** | |
| | **Slope** | **MSE** | **Slope** | **MSE** | | **Slope** | **MSE** | **Slope** | **MSE** |
| AdaBoost | 1.13 | 0.23 | 4.17 | 0.23 | CW-GBoost | 0.51 | 0.23 | 0.24 | 0.05 |
| CART | 0.53 | 0.25 | 0.57 | 0.23 | Extra Survival | 0.29 | 0.28 | 0.30 | 0.04 |
| CatBoost | 0.62 | 0.24 | 0.67 | 0.19 | Survival GBoost | 0.26 | 0.27 | 0.22 | 0.10 |
| C4.5 | 0.15 | 0.49 | 0.30 | 0.35 | LSVM | 0.06 | 0.04 | 0.36 | 0.03 |
| FIGS | 0.51 | 0.26 | 0.74 | 0.21 | NLSVM | 0.10 | 0.03 | 0.39 | 0.03 |
| GOSDT-GUESSES | 0.25 | 0.37 | 0.27 | 0.33 | Penalized Cox | 1.30 | 0.22 | 0.28 | 0.03 |
| GBoost | 0.80 | 0.24 | 2.09 | 0.23 | Survival RF | 0.06 | 0.05 | 0.28 | 0.04 |
| HS | 0.51 | 0.26 | 0.74 | 0.21 | Survival Trees | 0.10 | 0.34 | 0.20 | 0.08 |
| XGBoost | 0.45 | 0.28 | 0.55 | 0.24 | | | | | |

## Cohorts

The study uses data from three distinct cohorts to investigate mild cognitive impairment (*PD-MCI*) and subjective cognitive decline (*SCD*) in Parkinson's disease (PD). The cohorts are described in the following:

1. **Luxembourg Parkinson's Study** (**LuxPARK**, NCT05266872)[1]: This cohort includes individuals with PD who are participating in a longitudinal study that is focused on several different complications of PD, including cognitive decline. Since 2015, LuxPARK has recruited participants from Luxembourg and the surrounding "Greater Region" as part of the National Centre of Excellence in Research in Parkinson's Disease (NCER-PD). The study collects extensive clinical data, supporting a detailed assessment of motor and non-motor symptoms. Participants were selected based on a PD diagnosis by the UK Parkinson's Disease Society Brain Bank (UKPDSBB) criteria[2].
2. **Parkinson's Progression Markers Initiative** (**PPMI**, NCT04477785)[3]: The PPMI is a large-scale, multi-center study initiated in 2010 to identify biomarkers of Parkinson's disease progression (https://www.ppmi-info.org/). With approximately 50 international sites contributing to the cohort, PPMI includes patients with Parkinson's disease (PD) and healthy controls, thus enabling comparisons of cognitive outcomes across disease stages. To be included in the PPMI cohort, the presence of at least two of the following characteristics was required for the participants: resting tremor, bradykinesia, or rigidity, with either resting tremor or bradykinesia as essential criteria; or a single asymmetric resting tremor or asymmetric bradykinesia. The PPMI database offers a comprehensive range of clinical assessments, neuroimaging data, and biological samples, establishing it as a resource for investigating cognitive impairment in PD.
3. **French ICEBERG Cohort Study** (NCT02305147)[4]: The ICEBERG study, conducted at the Paris Brain Institute (Institut du Cerveau - ICM), is focused on identifying predictors factors of the onset and progression of patients with early-stage PD. The goal is to gain deeper insights into disease progression and to identify biomarkers for early detection and intervention. The ICEBERG study includes annual assessments over four years and is targeted to participants with a disease duration of three years or less at baseline. The diagnosis of PD is according to the UKPDSBB criteria. This cohort provides a variety of clinical assessments, including genetic and neuropsychological assessments, which contribute to a comprehensive understanding of the onset of PD.

The LuxPARK, PPMI, and ICEBERG cohorts adhere to ethical standards and have been approved by the relevant local ethics committees. Specifically, LuxPARK has been approved by the National Research Ethics Committee of Luxembourg (CNER Ref: 201407/13 and 202304/03). The ICEBERG study, sponsored by the French National Institute of Health and Medical Research (INSERM), was approved by the French local ethics committee (RCB: 2014-A00725-42). The PPMI study has received ethical approval from local ethics committees at all participating sites in the United States, Europe, and Australia. It complies with the Declaration of Helsinki and Good Clinical Practice (GCP) guidelines (see https://www.ppmi-info.org/about-ppmi/ppmi-clinical-sites for details). In all cohorts, all participants have provided written informed consent, ensuring ethical participation and data collection in each study.

The following inclusion criteria were used for this study:

1. A diagnosis of PD according to the UKPDSBB criteria for the LuxPARK and ICEBERG cohorts[2]. For the PPMI cohort, subjects had to have at least two of the following: rest tremor, bradykinesia, or rigidity, with either resting tremor or bradykinesia being one of the essential criteria or a single asymmetric resting tremor or asymmetric bradykinesia[3].
2. The clinically confirmed presence of mild cognitive impairment (*PD-MCI*) or subjective cognitive decline (*SCD*) interfering significantly with daily functioning within four years of

the baseline visit or the confirmed absence of these symptoms within the same period.

Participants in this study were classified using two measures of cognitive impairment: *PD-MCI* and *SCD*:

1. Objective cognitive impairment - mild cognitive impairment (*PD-MCI*):
   - *PD-MCI+*: Defined as participants with a Montreal Cognitive Assessment (MoCA) score <26.
   - *PD-MCI-*: Defined as participants with a MoCA score ≥26.
2. Subjective cognitive impairment - subjective cognitive decline (*SCD*):
   - *SCD+*: Defined as participants scoring Movement Disorder Society-United Parkinson's Disease Rating Scale (MDS-UPDRS) item 1.1 >1.
   - *SCD-*: Defined as participants scoring MDS-UPDRS item 1.1 ≤1.

The study used three distinct cohorts: LuxPARK, PPMI, and ICEBERG, which provided comprehensive data on participants diagnosed with PD from different regions and research initiatives. Each cohort contributed a set of baseline variables, and only those variables shared by all three cohorts were included in the analysis. This approach facilitated cross-cohort comparisons and strengthened the validity of the findings. These common variables included demographic and clinical assessment relevant to PD.

## Methods

This study used single-cohort and multi-cohort analyses to evaluate the predictive models for cognitive impairment in PD. Single-cohort analyses were conducted separately within each cohort (LuxPARK, PPMI, and ICEBERG) to assess model performance and identify cohort-specific predictive factors. Multi-cohort analyses included cross-cohort (using split training and validation data from all cohorts) and leave-one-cohort-out validation (training on two cohorts and testing on the third cohort), ensuring the generalizability of predictive models across diverse patient populations. Stratified data partitioning was applied to maintain proportional representation across training, testing, and validation sets. Further details on the data pre-processing, cross-validation procedure, feature selection, and machine learning (ML) approach evaluation are described below.

## Data preprocessing

Before the analysis, data aggregation was performed to ensure a comprehensive and indicative representation of each participant's clinical profile (see Supplementary Figure 18). This process involved combining related clinical variables into composite indicators that capture an integrated view of participant characteristics while reducing data dimensionality. Aggregated variables, as listed in Supplementary Table 15, included the summation of multiple MDS-UPDRS items (second column in the table) to create relevant summary measures.

For participants with missing items prior aggregation, missing values were replaced by averaging available items, as shown in the third column of the table. This approach retained clinical information and allowed for consistent and interpretable key predictors across single-cohort and multi-cohort analyses.
The average for the missing item for $i^{th}$ participant can be expressed as:

$$\text{missing } x_{ij} = \frac{\sum_{j=1}^{k} x_{ij}}{m_j}$$

where

$i = 1, \ldots, n$
$j = 1, \ldots, k$
$m_i$ = number of non-missing values for $i^{th}$ participant.

The $k$ is the number of variables used for variable aggregation, and $n$ is the total number of samples.

Subsequently, the aggregated variable was calculated as the summation of the items:

aggregated variable, $AV_i = \sum_{j=1}^{k} x_{ij}$

where $x_{ij}$ represents the individual items being aggregated for the $i^{th}$ participant.

**Supplementary Figure 15**. **Data processing and model development with nested cross-validation.** Workflow for data processing and model development, illustrating the process, including variable aggregation and the nested cross-validation workflow (Supplementary Figure 16) for model training and evaluation. Data processing steps, including missing values imputation, cross-study normalization, one-hot encoding, undersampling, and feature selection, were conducted independently on the training and validation sets to avoid data leakage, as detailed in the Supplementary Figure 17.

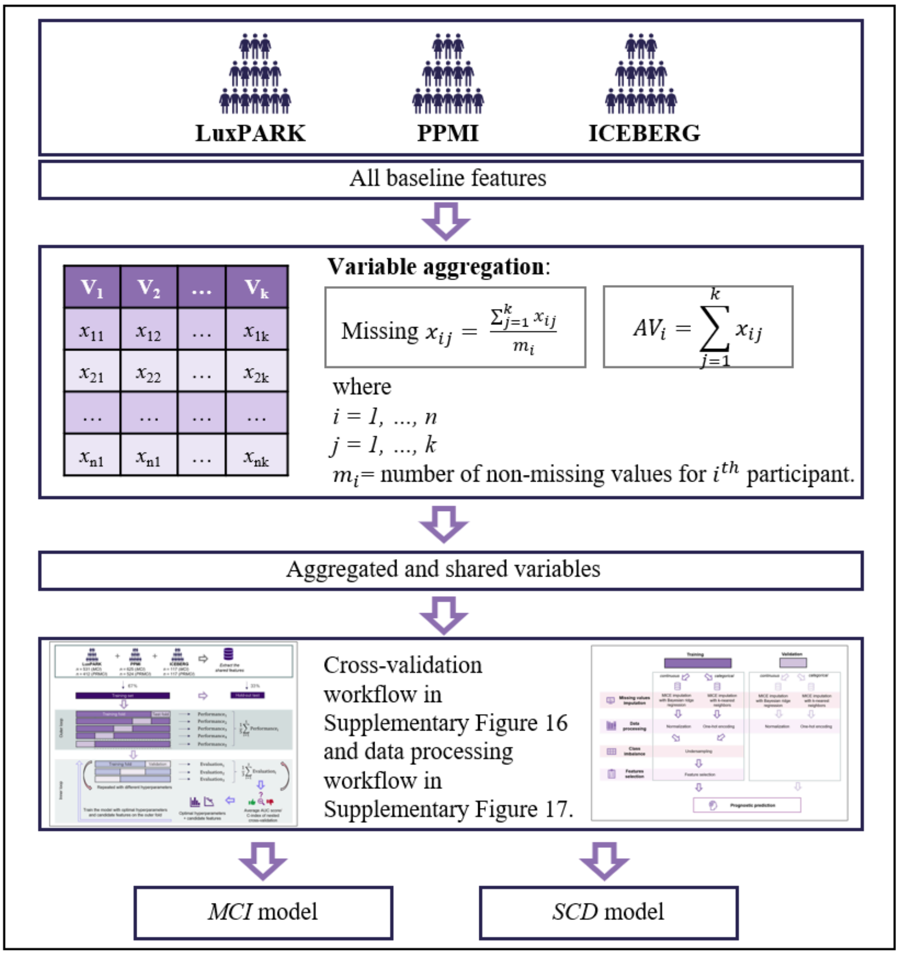

**Supplementary Table 15. Definition of aggregated feature variables from MDS-UPDRS.** The definition of aggregated feature variables derived from original MDS-UPDRS variables, where missing values were addressed by averaging non-missing related variables. Items retained for analysis were marked with an asterisk (*), ensuring that the data were representative and reducing the potential for bias.

| Aggregated variable | MDS-UPDRS items included | | Missing data strategy |
|---|---|---|---|
| Rigidity upper extremities | 3.3b | Rigidity - RUE | Average of items 3.3a to 3.3e |
| | 3.3c | Rigidity - LUE | |
| Rigidity lower extremities | 3.3d | Rigidity - RLE | Average of items 3.3a to 3.3e |
| | 3.3e | Rigidity - LLE | |
| Total rigidity | 3.3a | Rigidity - Neck | Average of items 3.3a to 3.3e |
| | 3.3b | Rigidity - RUE | |
| | 3.3c | Rigidity - LUE | |
| | 3.3d | Rigidity - RLE | |
| | 3.3e | Rigidity - LLE | |
| Bradykinesia score | 3.2 * | Facial expression | Average of items 3.2, 3.4 to 3.8, and 3.14 |
| | 3.4a | Finger tapping right hand | |
| | 3.4b | Finger tapping left hand | |
| | 3.5a | Hand movements - right hand | |
| | 3.5b | Hand movements - left hand | |
| | 3.6a | Pronation-supination - right hand | |
| | 3.6b | Pronation-supination - left hand | |
| | 3.7a | Toe tapping - right foot | |
| | 3.7b | Toe tapping - left foot | |
| | 3.8a | Leg agility - right leg | |
| | 3.8b | Leg agility - left leg | |
| | 3.14 * | Global spontaneity of movement | |
| Axial symptoms score | 2.12 * | Walking and balance | Average of items 2.12, 2.13, 3.1, 3.9, 3.11 to 3.13 |
| | 2.13 * | Freezing | |
| | 3.1 | Speech | |
| | 3.9 * | Arising from chair | |
| | 3.11 | Freezing of gait | |
| | 3.12 | Postural stability | |
| | 3.13 * | Posture | |
| Selective axial symptoms score | 2.12 * | Walking and balance | Average of items 2.12, 3.9, 3.12, 3.13 |
| | 3.9 * | Arising from chair | |
| | 3.12 | Postural stability | |
| | 3.13 * | Posture | |
| Freezing of gait | 2.13 * | Freezing | Average of items 2.13, 3.11 |
| | 3.11 | Freezing of gait | |
| Rest tremor score | 2.1 | Tremor | Average of items 3.17 |
| | 3.17a | Rest tremor amplitude - RUE | |
| | 3.17b | Rest tremor amplitude - LUE | |
| | 3.17c | Rest tremor amplitude - RLE | |
| | 3.17d | Rest tremor amplitude - LLE | |
| | 3.17e | Rest tremor amplitude - lip/jaw | |
| | 3.18 | Constancy of rest tremor | |
| Rest tremor amplitude score | 2.1 | Tremor | Average of items 3.17 |
| | 3.17a | Rest tremor amplitude - RUE | |
| | 3.17b | Rest tremor amplitude - LUE | |
| | 3.17c | Rest tremor amplitude - RLE | |
| | 3.17d | Rest tremor amplitude - LLE | |
| | 3.17e | Rest tremor amplitude - lip/jaw | |
| Tremor score | 2.1 | Tremor | Average of items 3.17 |
| | 3.15a | Postural tremor - right hand | |
| | 3.15b | Postural tremor - left hand | |
| | 3.16a | Kinetic tremor - right hand | |
| | 3.16b | Kinetic tremor - left hand | |
| | 3.17a | Rest tremor amplitude - RUE | |
| | 3.17b | Rest tremor amplitude - LUE | |
| | 3.17c | Rest tremor amplitude - RLE | |
| | 3.17d | Rest tremor amplitude - LLE | |
| | 3.17e | Rest tremor amplitude - lip/jaw | |
| | 3.18 | Constancy of rest tremor | |

## Cross-validation

The cross-validation framework used in this study is outlined in Supplementary Figure 16 and involves a structured workflow for both single-cohort and multi-cohort analyses. After extracting baseline variables common to all three cohorts and aggregating variables where appropriate, the data were divided into a training set (67% of the data) and a hold-out test set (33% of the data). One third of the data was randomly selected as a hold-out set for both single-cohort and multi-cohort analyses and the split was stratified by cohort (for the multi-cohort analysis) and outcome variable to ensure proportional representation of each cohort in all partitions (training, testing and external validation). The same proportion of data from each cohort was randomly selected for each partition. For single-cohort analyses, stratification was applied within each cohort independently to maintain a balanced outcome distribution. In addition, the test and validation sets only included patients who did not experience the event at baseline. This ensured that these sets were used to assess the model's ability to predict future outcomes and to avoid over-estimating performance by evaluating past events.

Within each training set, a 5-fold cross-validation, known as the outer loop, was applied to assess the average cross-validated predictive performance of the model. A 3-fold nested cross-validation (inner loop) was performed for each fold in this outer loop to optimize hyperparameters and select features. This inner loop ensured that the optimal combination of hyperparameters and corresponding candidate features that yielded the highest average predictive performance was selected. To ensure the integrity of the study and enhance the generalizability of the findings, participants with *PD-MCI*+ or *SCD*+ at baseline were excluded from the testing and hold-out test sets. This exclusion was necessary to prevent the incorporation of cases with pre-existing cognitive impairment, which could lead to an artificially optimistic predictive performance. This nested cross-validation aimed to improve model generalizability and robustness by leveraging hyperparameter tuning and feature selection for improved predictive capability across cohorts.

Data processing steps were performed independently in each cross-validation fold for the training, validation, and hold-out test sets to prevent data leakage, including the missing values imputation, cross-study normalization, categorical encoding, undersampling, and feature selection techniques (see Supplementary Figure 17):

**Missing value imputation**: Missing values in baseline features were handled using Multiple Imputation by Chained Equations (MICE)[5,6]. Bayesian ridge regression was used for continuous variables and k-nearest neighbors for categorical variables. Variables with more than 50% missingness were excluded from the analysis to ensure data quality. Imputation was specified to baseline characteristics and not extended to outcomes, thus ensuring the integrity of predictor-outcome relationships. This approach avoids introducing artificial relationships that could bias model performance metrics or indistinct true associations within the data.

**Cross-study normalization**: Continuous variables were standardized using cross-study normalization techniques, including mean-centering[7], standardization[7], quantile normalization[8,9], ComBat[10,11], Ratio-A[12], and M-ComBat[13], to enhance comparability across cohorts and maintain data consistency. In this study, the term "normalized" data refers to continuous variables that have undergone a transformation process with the objective of ensuring comparability across different cohorts. These normalizations serve to mitigate the effects of cohort-specific biases, thereby ensuring consistency and enhancing the robustness of multi-cohort analyses. In contrast, the term "unnormalized" data refers to continuous variables that have been left in their original state without undergoing any normalization process. This approach allows the data to retain the natural distribution and characteristics of each cohort without the need for standardization or adjustment for inter-cohort differences.

**Supplementary Figure 16. Machine learning workflow with 5-fold and nested 3-fold cross-validation.** Illustration of the machine learning and cross-validation workflow. The workflow involves training and evaluating machine learning models for cognitive impairment prognosis using 5-fold cross-validation to assess average performance. A 3-fold nested cross-validation on the training set data was used to optimize hyperparameters and select the most informative features.

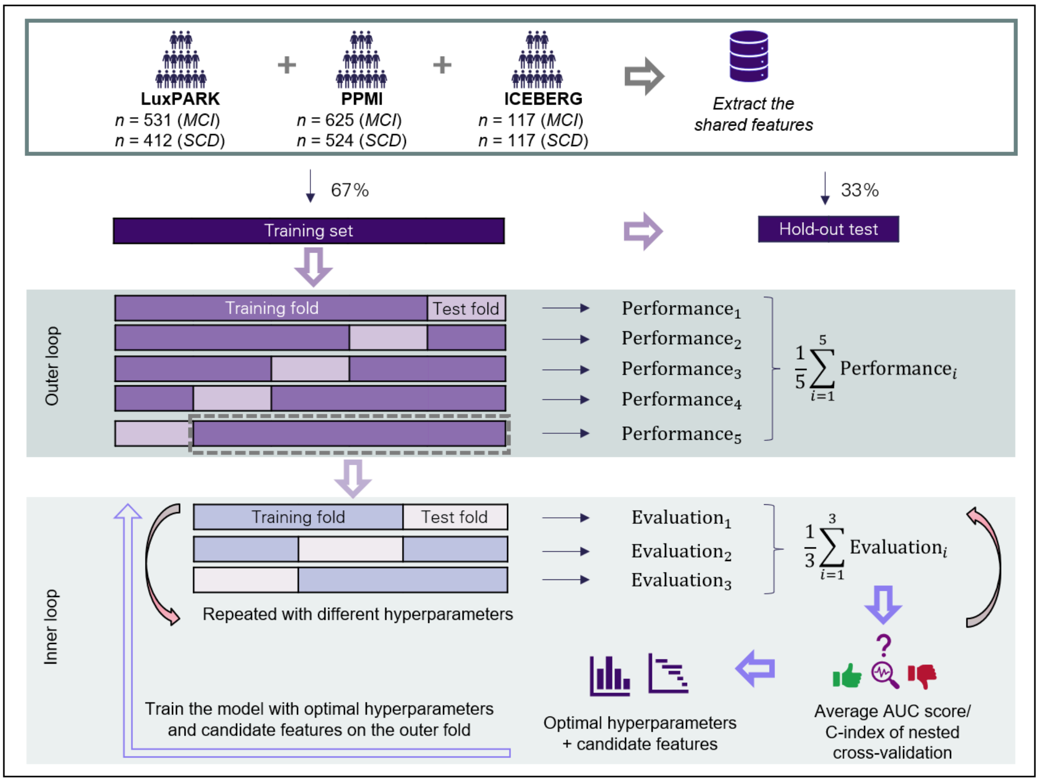


**Categorical encoding**: Categorical variables were transformed using one-hot encoding to ensure compatibility with ML algorithms[14], which typically require numerical input. This approach ensures that categorical data is accurately represented for ML algorithms, thus maintaining its integrity and interpretability.

**Undersampling**: To address outcome imbalance, undersampling was applied to the training set to ensure a balanced representation of outcomes during model training[15,16], whereby the majority class were selectively reduced. This approach ensured that the model was trained on a balanced representation of outcomes, thereby preventing any bias towards the majority class, and improved the model's sensitivity to minority outcomes, promoting more reliable and equitable predictions.

**Feature selection**: Recursive feature elimination (RFE) (which iteratively removes the least important features according to performance estimates), and bidirectional stepwise feature selection (which combines forward and backward selection to identify optimal feature combination) were used for feature selection in the nested cross-validation process[17,18]. Permutation importance scores were calculated for each model within each nested fold and during hyperparameter tuning[19], allowing identification of candidate features with significant predictive impact.

These data processing steps, implemented independently within each fold of the 2-level cross-validation, were important for robust model development. This approach ensures no data leakage while improving model performance and interpretability.

This study applied both ML classification and time-to-event techniques to identify predictors of *PD-MCI* and *SCD*. The predictive performance was assessed through the Area Under the Curve

(AUC) for classification and the concordance index (C-index) for time-to-event analyses. The algorithms used are described in detail below.

**Supplementary Figure 17. Data processing and analysis workflow for cross-validation cycles.** Overview of the data processing and analysis workflow applied during each cross-validation cycle to optimize and evaluate the prognostic models. The workflow covers multiple steps, including missing value imputation, cross-study normalization, one-hot encoding, undersampling, and feature selection.

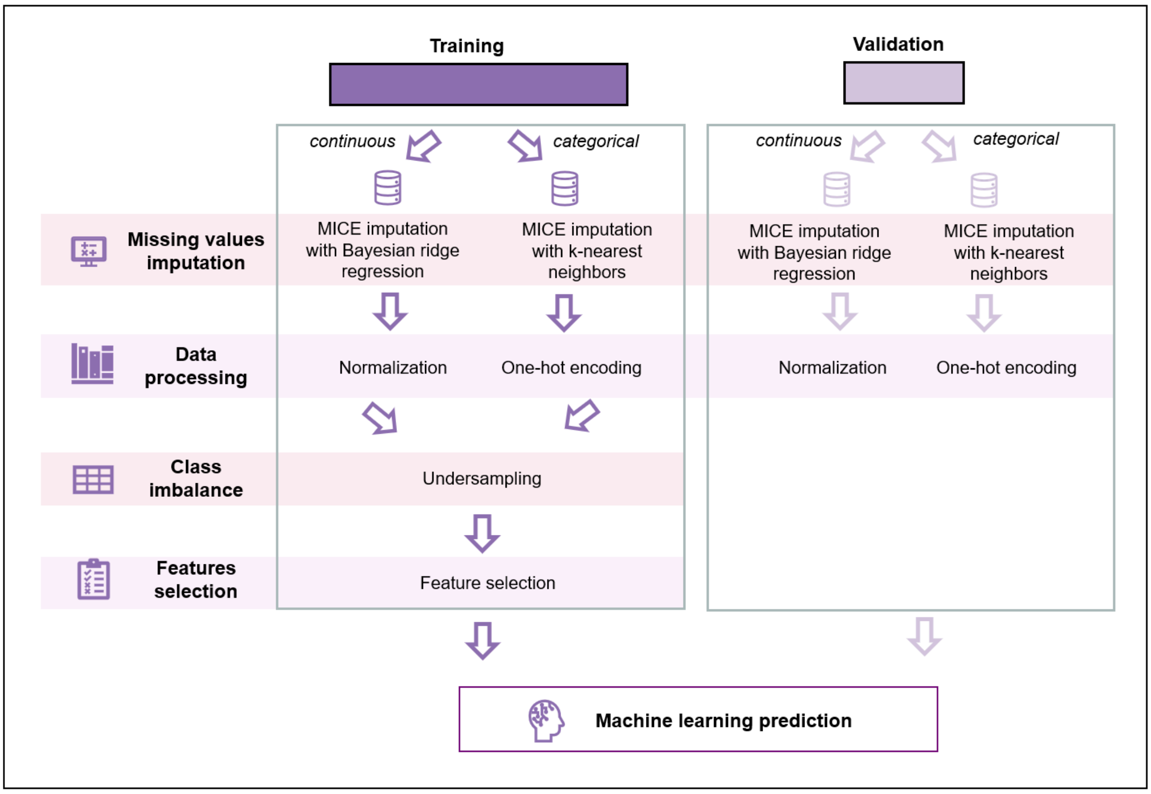


## Machine learning classification

In this study, nine classification algorithms were used to predict *PD-MCI* and *SCD*. Adaptive Boosting (AdaBoost) is a method that combines multiple weak models to form a more robust prediction[20], with a particular focus on correcting misclassifications. The Classification and Regression Trees (CART)[21] and C4.5 trees[22] provided simple decision trees that split the data to maximize outcome similarity within each group. Additionally, the C4.5 trees used pruning to prevent overfitting.

Category Boosting (CatBoost) effectively handles categorical data, ensuring consistent performance across diverse input types[23]. The Fast Interpretable Greedy-Tree Sums (FIGS) method emphasizes interpretability[24], whereby additive trees are constructed to highlight key features. The Fast-Sparse Decision Tree (GOSDT-GUESSES) generated models with minimal splits, balancing simplicity and accuracy[25]. Gradient Boosting (GBoost) refined predictions by iteratively minimizing errors[26], while Hierarchical Shrinkage (HS) reduced the number of features for greater interpretability[27]. Lastly, Extreme Gradient Boosting (XGBoost) introduced efficiency by incorporating regularization techniques, which prevent overfitting[28].

**Time-to-event analysis**

In the time-to-event analysis, eight ML algorithms were used to model the time-to-*PD-MCI* and time-to-*SCD*, considering the duration of the event occurrence and censoring. The component-wise gradient boosting (CW-GBoost) method adapts GBoost for use with time-to-event data[29], enabling predictions through a stepwise component-based approach. Survival Trees and Extra Survival Trees were used[30,31], resulting in the creation of decision trees that were specific to survival data. These trees segmented patients based on clinical features and optimized each branch for survival prediction. Furthermore, Survival Gradient Boosting (Survival GBoost) extended GBoost to prioritize accuracy in predicting the outcome[17].

In the case of linear approaches, the Survival Linear Support Vector Machine (LSVM) and Naive LSVM (NLSVM) used support vector principles to achieve optimal separation for the outcome over time[30,32]. The LSVM model incorporated additional optimizations specific to time-to-event analysis. The penalized Cox regression model introduced regularization to the traditional Cox model[33,34], thereby controlling for overfitting in high-dimensional data by penalizing less relevant predictors. Lastly, the Random Survival Forest (Survival RF) used an ensemble of survival trees to capture intricate interactions between variables[35], thereby providing robust prediction for the outcomes.

## References


1. Pavelka, L. *et al.* Luxembourg Parkinson's study -comprehensive baseline analysis of Parkinson's disease and atypical parkinsonism. *Front. Neurol.* **14**, 1330321 (2023).
2. Gibb, W. R. & Lees, A. J. The relevance of the Lewy body to the pathogenesis of idiopathic Parkinson's disease. *Journal of Neurology, Neurosurgery & Psychiatry* **51**, 745–752 (1988).
3. Marek, K. *et al.* The Parkinson Progression Marker Initiative (PPMI). *Progress in Neurobiology* **95**, 629–635 (2011).
4. Dodet, P. *et al.* Sleep disorders in Parkinson's disease, an early and multiple problem. *npj Parkinson's Disease* **10**, 46 (2024).
5. Azur, M. J., Stuart, E. A., Frangakis, C. & Leaf, P. J. Multiple imputation by chained equations: what is it and how does it work? *International Journal of Methods in Psychiatric Research* **20**, 40–49 (2011).
6. van Buuren, S. & Groothuis-Oudshoorn, K. mice: Multivariate Imputation by Chained Equations in R. *J. Stat. Softw.* **45**, 1–67 (2011).
7. Luo, J. *et al.* A comparison of batch effect removal methods for enhancement of prediction performance using MAQC-II microarray gene expression data. *The Pharmacogenomics Journal* **10**, 278–291 (2010).
8. Bolstad, B. M., Irizarry, R. A., Åstrand, M. & Speed, T. P. A comparison of normalization methods for high density oligonucleotide array data based on variance and bias. *Bioinformatics* **19**, 185–193 (2003).
9. Kostka, D. & Spang, R. Microarray Based Diagnosis Profits from Better Documentation of Gene Expression Signatures. *PLOS Computational Biology* **4**, e22 (2008).
10. Johnson, W. E., Li, C. & Rabinovic, A. Adjusting batch effects in microarray expression data using empirical Bayes methods. *Biostatistics* **8**, 118–127 (2007).
11. Chen, C. *et al.* Removing Batch Effects in Analysis of Expression Microarray Data: An Evaluation of Six Batch Adjustment Methods. *PLOS ONE* **6**, e17238 (2011).
12. Lazar, C. *et al.* Batch effect removal methods for microarray gene expression data integration: a survey. *Brief Bioinform* **14**, 469–490 (2013).
13. Stein, C. K. *et al.* Removing batch effects from purified plasma cell gene expression microarrays with modified ComBat. *BMC Bioinformatics* **16**, 63 (2015).
14. Dahouda, M. K. & Joe, I. A Deep-Learned Embedding Technique for Categorical Features Encoding. *IEEE Access* **9**, 114381–114391 (2021).
15. Rahman, M. M. & Davis, D. N. Cluster based under-sampling for unbalanced cardiovascular data. in *Proceedings of the World Congress on Engineering* 3–5 (2013).
16. Bach, M., Werner, A. & Palt, M. The Proposal of Undersampling Method for Learning from Imbalanced Datasets. *Procedia Computer Science* **159**, 125–134 (2019).

17. Karami, G., Giuseppe Orlando, M., Delli Pizzi, A., Caulo, M. & Del Gratta, C. Predicting Overall Survival Time in Glioblastoma Patients Using Gradient Boosting Machines Algorithm and Recursive Feature Elimination Technique. *Cancers* **13**, 4976 (2021).
18. Spooner, A. *et al.* A comparison of machine learning methods for survival analysis of high-dimensional clinical data for dementia prediction. *Scientific Reports* **10**, 20410 (2020).
19. Nirmalraj, S. *et al.* Permutation feature importance-based fusion techniques for diabetes prediction. *Soft Computing* (2023) doi:10.1007/s00500-023-08041-y.
20. Freund, Y. & Schapire, R. E. A Decision-Theoretic Generalization of On-Line Learning and an Application to Boosting. *Journal of Computer and System Sciences* **55**, 119–139 (1997).
21. Berk, R. A. Classification and Regression Trees (CART). in *Statistical Learning from a Regression Perspective* 129–186 (Springer International Publishing, Cham, 2016). doi:10.1007/978-3-319-44048-4_3.
22. Quinlan, J. R. Improved Use of Continuous Attributes in C4.5. *jair* **4**, 77–90 (1996).
23. Prokhorenkova, L., Gusev, G., Vorobev, A., Dorogush, A. V. & Gulin, A. CatBoost: unbiased boosting with categorical features. in *Advances in Neural Information Processing Systems 31 (NeurIPS 2018)* vol. 31 (2018).
24. Tan, Y. S. *et al.* Fast Interpretable Greedy-Tree Sums. (2022) doi:10.48550/arXiv.2201.11931.
25. McTavish, H. *et al.* Fast Sparse Decision Tree Optimization via Reference Ensembles. in *Proceedings of the AAAI Conference on Artificial Intelligence* 9604–9613 (2022).
26. Friedman, J. H. Greedy function approximation: A gradient boosting machine. *Ann. Stat.* **29**, 1189–1232 (2001).
27. Agarwal, A., Tan, Y. S., Ronen, O., Singh, C. & Yu, B. Hierarchical Shrinkage: improving the accuracy and interpretability of tree-based methods. *arXiv* (2022) doi:10.48550/ARXIV.2202.00858.
28. Chen, T. & Guestrin, C. XGBoost: A Scalable Tree Boosting System. in *Proceedings of the 22nd ACM SIGKDD International Conference on Knowledge Discovery and Data Mining* 785–794 (ACM, New York, NY, USA, 2016). doi:10.1145/2939672.2939785.
29. He, K. *et al.* Component-wise gradient boosting and false discovery control in survival analysis with high-dimensional covariates. *Bioinformatics* **32**, 50–57 (2016).
30. Bertsimas, D., Dunn, J., Gibson, E. & Orfanoudaki, A. Optimal survival trees. *Mach. Learn.* **111**, 2951–3023 (2022).
31. Geurts, P., Ernst, D. & Wehenkel, L. Extremely randomized trees. *Mach. Learn.* **63**, 3–42 (2006).
32. Wang, M. *et al.* Dementia risk prediction in individuals with mild cognitive impairment: a comparison of Cox regression and machine learning models. *BMC Med Res Methodol* **22**, 284 (2022).
33. Park, M. Y. & Hastie, T. *L*1-regularization path algorithm for generalized linear models. *J. R. Stat. Soc. Series B Stat. Methodol.* **69**, 659–677 (2007).
34. Simon, N., Friedman, J., Hastie, T. & Tibshirani, R. Regularization Paths for Cox's Proportional Hazards Model via Coordinate Descent. *J Stat Softw* **39**, 1–13 (2011).
35. Ishwaran, H., Kogalur, U. B., Blackstone, E. H. & Lauer, M. S. Random survival forests. *Ann. Appl. Stat.* **2**, 841–860 (2008).
